# Diversity and evolution of sex determination systems in terrestrial isopods

**DOI:** 10.1038/s41598-017-01195-4

**Published:** 2017-04-24

**Authors:** Thomas Becking, Isabelle Giraud, Maryline Raimond, Bouziane Moumen, Christopher Chandler, Richard Cordaux, Clément Gilbert

**Affiliations:** 1 Université de Poitiers, UMR CNRS 7267 Ecologie et Biologie des Interactions, Equipe Ecologie Evolution Symbiose, TSA 51106, 86073 Poitiers Cedex 9, France; 2 Department of Biological Sciences, SUNY Oswego, Oswego, New York 13126 USA

## Abstract

Sex determination systems are highly variable in many taxa, sometimes even between closely related species. Yet the number and direction of transitions between these systems have seldom been characterized, and the underlying mechanisms are still poorly understood. Here we generated transcriptomes for 19 species of terrestrial isopod crustaceans, many of which are infected by *Wolbachia* bacterial endosymbionts. Using 88 single-copy orthologous genes, we reconstructed a fully resolved and dated phylogeny of terrestrial isopods. An original approach involving crossings of sex-reversed individuals allowed us to characterize the heterogametic systems of five species (one XY/XX and four ZW/ZZ). Mapping of these and previously known heterogametic systems onto the terrestrial isopod phylogeny revealed between 3 and 13 transitions of sex determination systems during the evolution of these taxa, most frequently from female to male heterogamety. Our results support that WW individuals are viable in many species, suggesting sex chromosomes are at an incipient stage of their evolution. Together, these data are consistent with the hypothesis that nucleo-cytoplasmic conflicts generated by *Wolbachia* endosymbionts triggered recurrent turnovers of sex determination systems in terrestrial isopods. They further establish terrestrial isopods as a model to study evolutionary transitions in sex determination systems and pave the way to molecularly characterize these systems.

## Introduction

In metazoans, sex determination enables the development of individuals as males or females. Sex determination is widespread and, thus, could be expected to be governed by highly conserved mechanisms throughout metazoan evolution. While the bottom ends of sex determination pathways indeed tend to be conserved^1^, the higher steps show extensive evolutionary flexibility, as they can be triggered by a variety of mechanisms^2^. Genetic Sex Determination (GSD), in which the signal inducing sex differentiation is carried by sex chromosomes, is the most common mechanism in metazoans. In GSD, sex is determined by a locus that is heterozygous in one sex (the heterogametic sex) and homozygous in the other sex (the homogametic sex)^2^. A variety of GSD systems exist and several approaches have been developed to characterize them directly or indirectly. Most of these approaches rely on the presence of differences in size and/or morphology in the sex chromosomes of the heterogametic sex (heteromorphy). Such differences are expected to result from the degeneration of the sex chromosome present only in the heterogametic sex, which, after gaining a master sex determination gene, ceases to recombine at meiosis^3^. Many studies have shown that the primary signal of sex determination can be subject to quick and frequent changes during evolution^4–6^. Moreover, in several taxa, sex determination mechanisms do not match phylogenetic relationships, sometimes even in groups of closely related species^7–9^. This indicates that sex chromosome turnovers can take place recurrently in some lineages.

Several evolutionary forces have been proposed to explain turnovers of sex determination mechanisms: sexually antagonistic selection^10^, accumulation of deleterious mutations in the heterogametic sex chromosome^11^, the combination of these two effects known as the “hot potato model”^12^, meiotic drive^13^, heterozygote advantage^14^ and sex ratio selection^15, 16^. In particular, sex ratio selection occurs to restore balanced sex ratios in animals producing progenies that are skewed towards one sex^17^. Sex ratio biases can be caused by sex ratio distorters, which are selfish genetic elements with sex-biased inheritance that enhance their own transmission by biasing sex ratios to their advantage.

One example of sex ratio distorters are *Wolbachia* bacterial endosymbionts. *Wolbachia* are strictly intracellular, maternally-inherited, α-proteobacteria that are widespread in arthropods and some nematodes^18^. In arthropods, *Wolbachia* often enhances its own transmission by manipulating host reproduction in favor of infected females, which can have important consequences on the evolution of host sex determination mechanisms^19^. This is well illustrated by the common pillbug *Armadillidium vulgare* ^19, 20^. In this terrestrial isopod species, GSD follows female heterogamety (ZW females and ZZ males). However, some females have been shown to produce progenies that are highly biased towards females. This is due to the presence of feminizing *Wolbachia*, which induce feminization of (non-transmitting) genetic males into (transmitting) phenotypic females^21, 22^. In *A*. *vulgare* lines in which *Wolbachia* are present, the W sex chromosome ends up being lost. As a result, all individuals are ZZ genetic males, those carrying *Wolbachia* develop as females whereas uninfected individuals develop as males. As the *Wolbachia* transmission rate from mother to offspring is ~90%, progenies are composed of ~90% females and ~10% males. This is a perfect example of cytoplasmic sex determination^19, 20^.

*Wolbachia*-mediated sex ratio distortion induces nucleo-cytoplasmic conflicts, leading to strong selective pressures promoting the evolution of nuclear repressors of feminization that can restore balanced sex-ratios, i.e. masculinizing genes^23^. Such nuclear repressors may evolve as new male sex-determining genes, thereby establishing new male heterogametic systems XY/XX^24^. In addition, we recently showed that *Wolbachia* endosymbionts triggered the evolution of a new W sex chromosome in *A*. *vulgare* by horizontal genome transfer and nuclear incorporation as sex-determining factor^25^. Then, *A*. *vulgare* appears to experience frequent turnovers of sex-determining factors ultimately caused by feminizing *Wolbachia* endosymbionts^19, 26^. In this context, the widespread distribution of *Wolbachia* infection in terrestrial isopods^27, 28^ raises the possibility that *Wolbachia* endosymbionts may have impacted sex chromosome evolution in terrestrial isopods in general.

If *Wolbachia* endosymbionts triggered transitions between sex determination systems, it can be predicted that various heterogametic systems should be observed in diverse species and they should display a patchy distribution in the terrestrial isopod phylogeny. Interestingly, both female (ZW/ZZ) and male (XY/XX) heterogametic systems have been reported in various terrestrial isopod species^20^. However, transitions of sex chromosomes are difficult to establish due to the lack of a robust phylogenetic framework. Two studies have so far attempted to reconstruct molecular phylogenies of some terrestrial isopod species^29, 30^, but both yielded poorly resolved trees owing to the small number of markers used and the relatively low amount of phylogenetic signal contained in these markers. Here, we investigated the diversity and evolution of sex determination systems in terrestrial isopods in two steps: (i) we reconstructed the first fully-resolved molecular phylogeny of terrestrial isopods, and (ii) we assessed heterogametic systems in terrestrial isopod species using experimental procedures.

**Figure 1:**
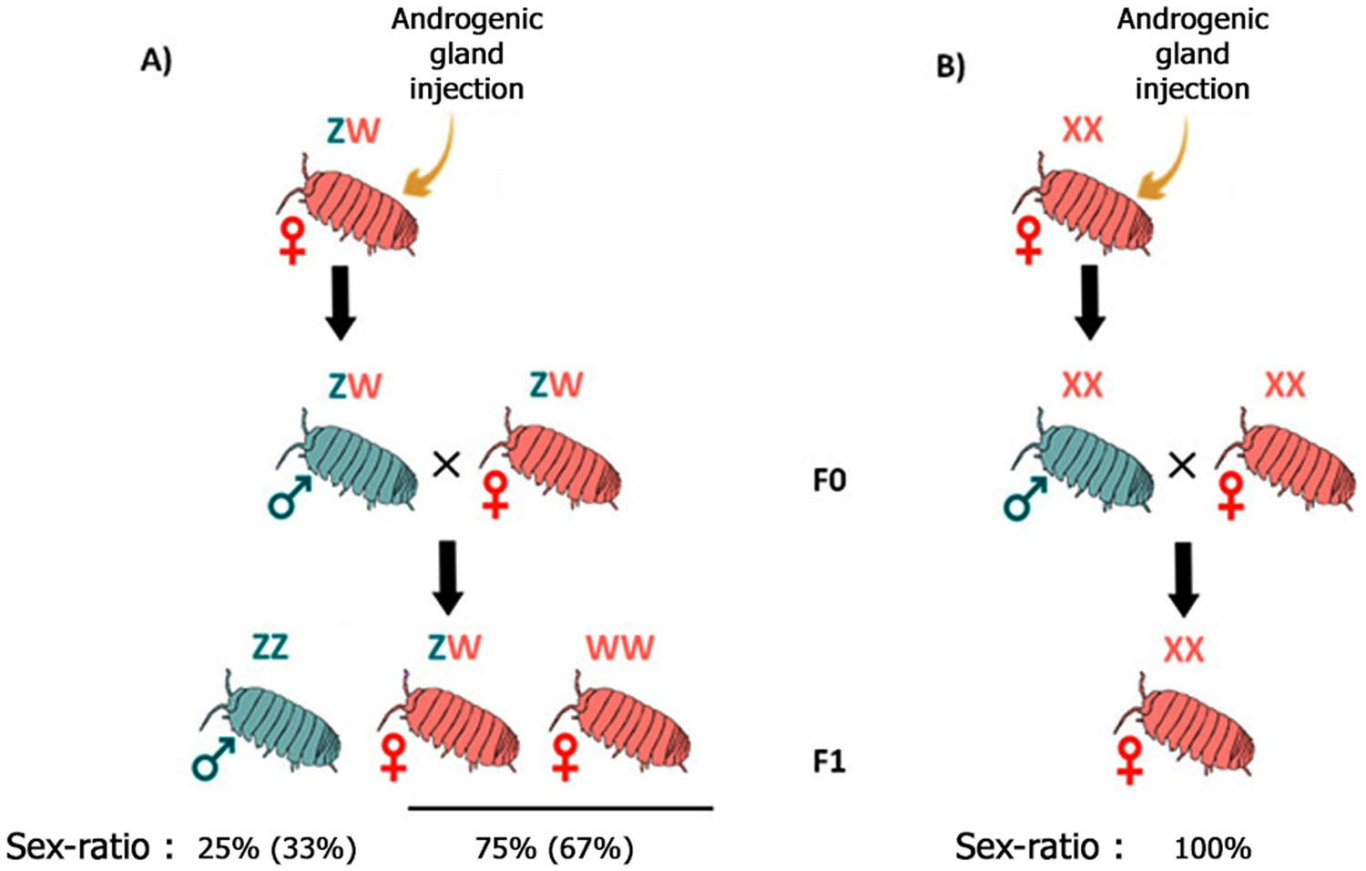
Principle of crossing experiments between neo-males (i.e. sex-reversed females) and genetic females. (**A**) Female heterogametic system (ZZ/ZW). The expected sex-ratio of the progeny of a neo-male and a genetic female is 25% males (ZZ) and 75% females (ZW and WW) if the WW genotype is viable, or 33% males (ZZ) and 67% females (ZW only) if the WW genotype is not viable. (**B**) Male heterogametic system (XX/XY). The expected sex-ratio of the progeny of a neo-male and a genetic female is 100% females (XX).

Classically, characterization of heterogametic systems can be achieved by combining cytogenetic methods with fluorescent *in situ* hybridization, possibly associated with chromosome mapping. However, cytogenetic analyses are often difficult in isopods and they may be uninformative in the case where sex chromosomes are homomorphic. Alternatively, comparative genomics approaches have recently been developed, often relying on mapping/coverage analysis of both male and female genomes^31–35^. However, these methods require large amounts of sequence data and crustacean genomes are often very large^36^. To circumvent these issues, we used an original experimental strategy based on a combination of sex reversals and crossings of individuals with identical sex chromosome genotypes^37^. These crossings are possible because of two features characterizing the peculiar sexual differentiation of terrestrial isopods: (i) individuals are sexually undifferentiated at birth and sexual differentiation occurs in a specific time window after birth^38, 39^, and (ii) male sexual differentiation is under the control of the androgenic hormone secreted by the male-specific androgenic gland^40, 41^. Then, it is possible to induce genetic females to differentiate as phenotypic males (termed “neomales”) by experimental implantation of androgenic glands during early sexual differentiation. These neomales are then crossed with their sisters, which is equivalent to crossing individuals with the same sex chromosome genotype. Analysis of the sex ratios of the resulting progenies allows definitive characterization of heterogametic systems (Fig. 1). This strategy enabled us to map heterogametic systems onto a robust phylogenetic context and infer transitions of sex chromosome systems during terrestrial isopod evolution.

## Results

### Phylogenetic analyses

**Figure 2:**
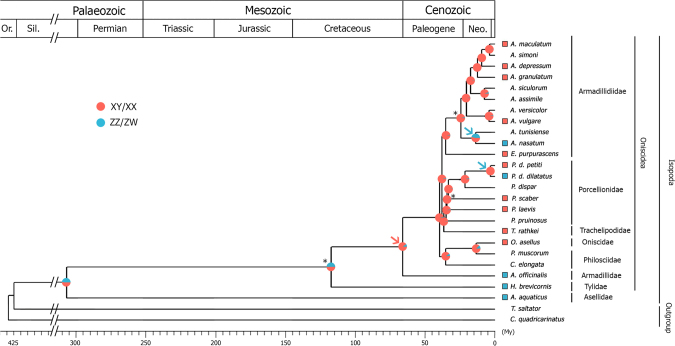
Ancestral state reconstruction of sex determination systems in terrestrial isopod crustaceans, based on a Bayesian phylogenetic analysis using 88 molecular markers. The reconstruction is based on the one-parameter equal rates model (“ER” model), considering the *A*. *assimile* sex determination system as unknown. Pie charts on nodes show the probability of the ancestral state at each node, calculated from 1,000 stochastic mappings. Branch length is scaled to time, with nodal ages corresponding to median posterior estimates. *Denote the calibration points. Red and blue squares at branch tips indicate a ZZ/ZW and XY/XX sex determination systems, respectively. Transitions of sex determination systems determined by parsimony ancestral state reconstruction are outlined with arrows (one red arrow for a transition from XY/XX system to ZZ/ZW, and two blue arrows for transitions from ZZ/ZW to XX/XY systems). Isopod family names and higher taxonomic ranks are shown on the right.

To reconstruct the phylogeny of terrestrial isopods, we generated transcriptome data for 19 species available in our laboratory and used the transcriptomes of 5 other relevant crustaceans that were publicly available (Supplementary Table S1). Our search for orthologous genes in the transcriptomes of 24 crustacean species yielded 88 markers (81 nuclear and 7 mitochondrial). Out of 22 isopod species, we found 76–88 markers in all species but *Helleria brevicornis* (67 markers) and *Asellus aquaticus* (66 markers). In addition, all 88 markers were found in the two outgroup species *Cherax quadricarinatus* (Australian freshwater crayfish) and *Talitrus saltator* (European sand hopper). When combined, these markers produced a 69,570 bp-long alignment with a very low amount of missing data (7%). Two subspecies for which the heterogametic system is known were not included in our RNA-seq experiment: *Porcellio dilatatus dilatatus* (XY/XX)^42^ and *Porcellio dilatatus petiti* (ZZ/ZW)^43^. To include these species in our phylogeny at lower cost, we first performed phylogenetic analyses of each of the 88 markers independently. Among the 35 markers that independently produced a topology identical to the one retrieved with the combined alignment, we selected the 10 longest ones (5 nuclear and 5 mitochondrial) and Sanger-sequenced them in the two *P*. *dilatatus* subspecies. The 10 markers sequenced in *P*. *d*. *dilatatus* (total length: 3,054 bp) and *P*. *d*. *petiti* (total length: 3,284 bp) were analyzed both separately as a combined 10-marker alignment and together with the other 78 markers. The phylogenetic analysis based on the 88-marker alignment produced a fully resolved tree, with all nodes supported by bootstrap values of 100% and all Bayesian posterior probabilities equal to 1 (Fig. 2, Supplementary Figure S1). The 10-marker tree was fully congruent with the 88-marker tree and strongly supported as well (all bootstrap values but one ≥95%, Supplementary Figure S1). We also compared the topology of the tree obtained using a combined alignment of the mitochondrial markers only (7 out of 88; alignment length: 5,847 bp) to that obtained with a combined alignment of the nuclear markers only (81 out of 88; alignment length: 63,723 bp). The two topologies were identical with each other and with the 88-marker and 10-marker trees (10 out of 88; alignment length: 10,773 bp), and they were both strongly supported (bootstrap scores for mitochondrial markers: all but two ≥95%; for nuclear markers: all but one ≥95%, Supplementary Figure S1).

Our phylogenetic analyses confirmed the monophyly of the Armadillidiidae family (all species from genus *Armadillidium* and *Eluma purpurascens*). The Porcellionidae family (all species from genus *Porcellio* and *Porcellionides pruinosus*) also appeared to be monophyletic, and was sister to the Trachelipodidae family (*Trachelipus rathkeii* in the phylogeny). By contrast, our analysis revealed the paraphyly of the Philosciidae family (*Philoscia muscorum* and *Chaetophiloscia elongata*), as it included *Oniscus asellus*, which belongs to the Oniscidae family. Finally, our analysis provided strong support for the position of the Tylidae family (*H*. *brevicornis* in the phylogeny) as sister to all other terrestrial isopods (Oniscidea suborder) (Fig. 2, Supplementary Figure S1).

### Divergence times

Estimations of divergence times suggested that terrestrial isopods (Oniscidea) diverged from the aquatic isopod suborder Asellota (*A*. *aquaticus*) between the late Carboniferous (corresponding to the explosion of terrestrial arthropod diversity^44, 45^) and the early Permian (307 My, 95% Highest Posterior Density (HPD): 213–362 My, Fig. 2 and Supplementary Figure S2). As expected, the estimated age of the *Armadillidium* and *Porcellio* genera, and of the Oniscidea suborder fit with the age of their respective oldest known fossils used as calibration points (25 My, 95% HPD: 23–27 My; 35 My, 95% HPD: 34–37 My and 117 My, 95% HPD: 110–155 My, respectively, Fig. 2 and Supplementary Figure S2). According to our analyses, the Trachelipodidae family diverged from other terrestrial isopods during the late Eocene (38 My, 95% HPD: 35–42 My, Fig. 2 and Supplementary Figure S2). The *Oniscus* genus diverged from the *Philoscia* genus during the Miocene (15 My, 95% HPD: 12–19 My, Fig. 2 and Supplementary Figure S2). The paraphyletic family Philosciidae originated between the late Eocene and the early Oligocene (36 My, 95% HPD: 32–48 My, Fig. 2 and Supplementary Figure S2). Finally, the *Armadillo* genus diverged from other terrestrial isopods during the late Cretaceous (66 My, 95% HPD: 57–79 My, Fig. 2 and Supplementary Figure S2).

### Substitution rates

Mitochondrial and nuclear codon alignments were used to estimate the rate of silent substitutions across isopods. When scaled to the fossil-calibrated chronogram, substitution rates in isopods were estimated at 3.89 × 10^−9^ and 4.17 × 10^−9^ silent substitutions per year for mitochondrial and nuclear sequences, respectively (Supplementary Figure S3). We also calculated the rate of non-synonymous/synonymous substitutions (dN/dS) for the two datasets. Both mitochondrial and nuclear dN/dS ratios were <1, thus indicating that our markers evolve under strong purifying selection (mitochondrial dN/dS = 0.28; nuclear dN/dS = 0.07).

### Assessment of heterogametic systems

Injections of androgenic glands were attempted in 135 young females from 15 terrestrial isopod species, representing 4–18 females per species (Supplementary Table S2). We discarded 25 individuals for which sex reversal was not successful (from 0 to 4 individuals per species) because they developed as females. In addition, 60 individuals died during the experiments (from 0 to 8 individuals per species). The remaining 50 individuals from 13 species developed as putative neomales (none was obtained for *P*. *muscorum* and *C*. *elongata*). Each putative neomale was crossed with 2 to 3 sisters for a total of 125 crosses, representing 2 to 18 crosses per species. We obtained 43 progenies from 11 species (none was obtained for *Armadillidium tunisiense* and *P*. *pruinosus*), representing 1–8 progenies per species (Supplementary Table S2).

Among the 43 progenies, 5 were discarded because the mothers were infected by *Wolbachia* (Supplementary Table S3). We also discarded 11 progenies because observed sex ratios did not significantly differ from a balanced sex ratio. This may be explained by small progeny sizes resulting in lack of statistical power and/or by the fact that individuals selected for sex reversal were undifferentiated young males instead of females. As a result, crosses occurred between genetic males (instead of neomales) and genetic females, hence the balanced sex ratio in the progenies. In sum, we obtained 27 progenies showing unbalanced sex ratios in the absence of *Wolbachia* infection from 7 species, representing 1–6 progenies per species (Supplementary Table S3).

**Figure 3:**
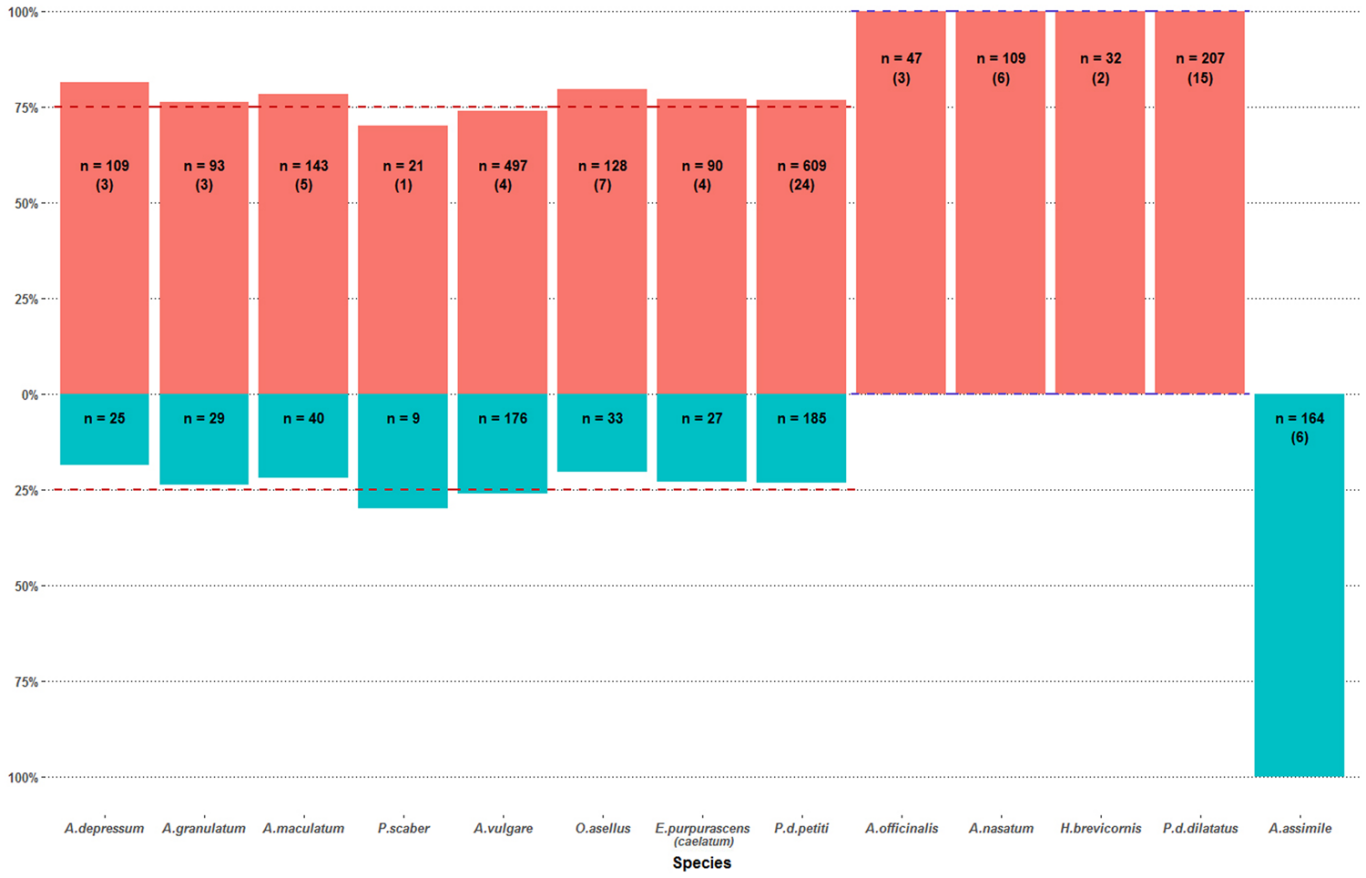
Sex ratio of progenies of 13 terrestrial isopod species resulting from crosses between genetic and sex-reversed females (neomales). Red and blue colors indicate the percentages of females and males, respectively. Red dashed lines indicate a 1/4♂: 3/4♀ sex ratio (expected in ZZ/ZW systems) and blue dashed lines indicate a 0♂: 1♀ sex ratio (expected in XY/XX systems). *n* is the number of pooled individuals per sex, and the number of corresponding progenies are specified in brackets for each species.

For each species for which we obtained at least two progenies, χ² tests indicated consistent sex ratio deviations among progenies. Therefore, we pooled data from these progenies and calculated overall sex ratios for each species (Fig. 3, Supplementary Table S4). Four species showed sex ratios indicating a female heterogametic system (ZZ/ZW): *Armadillidium depressum*, *Armadillidium granulatum*, *Armadillidium maculatum* and *Porcellio scaber*. Interestingly, for the three *Armadillidium* species, sex ratios were statistically different from 1/3♂:2/3♀ but not from 1/4♂:3/4♀, suggesting that the WW genotype is viable in these species (Supplementary Table S4). For *P*. *scaber*, the sex ratio was consistent with both 1/3♂:2/3♀ and 1/4♂:3/4♀, which prevented any conclusion to be drawn about viability of the WW genotype. This lack of resolution is probably attributable to the fact that we obtained only 30 offspring for this species. In addition, 2 species showed sex ratios indicating a male heterogametic system (XY/XX): *Armadillidium nasatum* and *Armadillo officinalis*. Our results for *A*. *nasatum* confirmed earlier findings^46^ and showed our approach is robust and reproducible. Finally, the sex ratio observed in *Armadillidium assimile* (100%♂:0%♀) cannot readily be explained by our predictions (Fig. 1), and further investigations are required to fully understand these results.

A review of the literature indicated that the heterogametic system has previously been assessed in 6 other terrestrial isopod species using sex reversal experiments^37, 42, 43, 47^ (Supplementary Table S3). To further analyze the data produced by these studies, we performed χ² tests, which were not systematically conducted at the time. All tests indicated consistent sex ratio deviations among progenies for each species. Therefore, we pooled data from these progenies and calculated overall sex ratios for each species (Fig. 3, Supplementary Table S4). We confirmed the original conclusions of a female heterogametic system (ZZ/ZW) in 4 species: *O*. *asellus* and *E*. *purpurascens* ^37^, *P*. *d*. *petiti* ^43^ and *Armadillidium vulgare* ^47^. Our statistical results indicated that the WW genotype is apparently viable in all four species. This confirmed earlier findings established after crossing F1 females (WW) with genetic males (ZZ) and analyzing F2 sex ratios under the prediction that such crossings should entirely be composed of females (ZW)^37, 47^. The same approach demonstrated that *P*. *d*. *petiti* has a viable WW genotype^43^. We also confirmed that *H*.*brevicornis* and *P*. *d*. *dilatatus* have a male heterogametic system (XX/XY).

### Transitions of heterogametic systems in terrestrial isopods

In addition to the 13 terrestrial isopod species discussed in the previous section, the heterogametic system has been determined using cytogenetics in the terrestrial isopods *Porcellio laevis* (ZZ/ZW)^48^ and *T*. *rathkei* (ZZ/ZW)^49^, as well as in a few aquatic isopods, including *A*. *aquaticus* (XY/XX)^50^. Mapping of sex determination systems onto the terrestrial isopod phylogeny revealed a heterogeneous distribution, which cannot readily be explained by a simple evolutionary scenario involving a single transition between male and female heterogametic systems. Despite missing information for 9 species (including *A*. *assimile* and 8 out of 24 ingroup species in our phylogenetic tree), the MESQUITE software^51^ parsimoniously identified that at least 3 transitions are required to explain the distribution of these sex determination systems (Fig. 2): one transition from male to female heterogamety (in the ancestor of a clade comprising the Armadillidiidae, Porcellionidae, Trachelipodidae, Oniscidae and Philoscidae families) and two transitions from female to male heterogamety (between the two subspecies *P*. *d*. *dilatatus* (XY/XX) and *P*. *d*. *petiti* (ZZ/ZW) and within the Armadillidiidae family). A fourth transition is required if *A*. *assimile* sex determination mechanism is considered as a new and independent system (in the ancestor of a clade grouping *Armadillidium siculorum* and *A*. *assimile* or later on the branch leading to *A*. *assimile* if *A*. *siculorum* is ZZ/ZW). In a separate analysis, a maximum likelihood-based reconstruction of ancestral states yielded between 11 and 13 transitions, depending on the model and the type of discrete states used (Supplementary Table S5). Both parsimony and maximum likelihood-based methods indicated that transitions from female to male heterogamety are twice as frequent as transitions from male to female heterogamety (Supplementary Table S5).

## Discussion

### Phylogenetic analyses

In this study, we used a large combination of 88 nuclear and mitochondrial molecular markers obtained by transcriptome sequencing to establish a fully resolved molecular phylogeny of terrestrial isopods (Supplementary Figure S1). We also identified a subset of 10 markers which produced the same topology with strong bootstrap support. We designed degenerate primers for these 10 loci and we showed they can be used to complement the phylogeny with additional species. As obtaining transcriptomes for a large number of species may be costly and/or time-consuming, we expect that the cheap and fast alternative 10-marker strategy we propose may enable further phylogenetic analyses of terrestrial isopods. Oniscidea being one of very few crustacean groups adapted to terrestrial lifestyle, their study provides unique research opportunities in fields such as ecology, ecophysiology and evolution^52^, which will be facilitated by the establishment of an adequately resolved phylogeny.

Our fully resolved phylogeny confirmed the monophyly of the Armadillidiidae and Porcellionidae families. These two taxa were considered as sister families^29^. However, this earlier study did not include a member of the Trachelipodidae family, which appears to be sister to the Porcellionidae, according to our phylogeny. Our results also highlight the intriguing evolution of *H*. *brevicornis*. As the only recognized species of the Helleriinae subfamily (grouped with the *Tylos* genus in the Tylidae family), this endemic species from the North Tyrrhenian area^53^ has been considered as a branch that diverged early during terrestrial isopod evolution based on morphological characters^54^. Characterized by a high mitochondrial AT nucleotide content, the early divergence of *H*. *brevicornis* from the other Oniscidea was already suspected^29^, without being able to solve the phylogenetic relationships of this species. Our work offers a definitive conclusion by confirming earlier molecular and morphological studies (Fig. 2). Our results also show that the Philosciidae family is paraphyletic, because of the clustering of *O*. *asellus* (Oniscidae) with *C*. *elongata* and *P*. *muscorum*. The close relationship between these two families has been suggested^30, 55^ but their respective monophyly could not be tested because of insufficient species sampling. This close relationship is congruent with morphological analyses, as both families share 5 pairs of similar pleopodal lungs covered with a slightly wavy membrane, a key character in the evolution of respiratory structures^56^. However, there is currently no obvious key morphological characteristic common to all Philosciidae species, which is a family presenting one of the highest species richness among terrestrial isopods and the richest family in terms of genera^55^. The unambiguous paraphyly of Philosciidae reported here calls for a reevaluation of the taxonomy of this family, which could involve its subdivision in several smaller families.

### Divergence times and estimates of substitution rates

Unlike insects, terrestrial isopods do not possess a waxy epicuticle, thus complicating the fossilization process^57^. The terrestrial isopod fossil record is thus relatively poor, implying that the divergence times of these taxa must be interpreted with caution. Our results differ from those obtained in an earlier study^58^, which included several terrestrial isopods as part of an investigation of the evolution of deep sea isopods. This discrepancy is likely due to the fact that they did not calibrate any node within terrestrial isopods^58^. Thus, our analyses are more likely to be closer to the true divergence times for this clade.

Our study also allowed us to calculate absolute silent substitution rates (dS) in terrestrial isopods, which are very similar between mitochondrial and nuclear sequences (3.89 × 10^−9^ and 4.17 × 10^−9^ silent substitutions per years, respectively). In comparison, several studies based on mitochondrial COI sequences from crustacean species showed substantial dS variations depending on the studied order. Whereas mitochondrial dS rates similar to those reported here were found in copepods (9.3 × 10^−9^, *Tigriopus* genus^59^) and aquatic isopods (4.6 × 10^−9^, *Stenasellus* genus^60^), it has been established that this rate might be 20 times higher in decapods (86 × 10^−9^ in genus *Alpheus* ^61^) for the same sequence (COI). More surprisingly, mitochondrial genes usually evolve at an elevated rate compared to nuclear genes, e.g. 11 to 62-fold higher in mammals and 1.4 to 18-fold higher in insects^62, 63^. By contrast, we did not observe such a variation in our datasets where mitochondrial and nuclear dS appear to be similar. To some extent, such a situation has already been reported in corals^64^, fungi^65^ and sponges^66^. In these taxa, a specific mitochondrial DNA repair function could be responsible for the low levels of mitochondrial divergence. In spite of the availability of well assembled mitochondrial genomes, no such gene is known in terrestrial isopods^67, 68^ and at present, we cannot explain why mitochondrial and nuclear dS are similar.

### Diversity of sex determination systems

We used an original strategy combining sex reversals and crossings of individuals with the same sex chromosome genotype to characterize heterogametic systems in 5 species of terrestrial isopods, including 4 species showing female heterogamety (*A*. *depressum*, *A*. *granulatum*, *A*. *maculatum* and *P*. *scaber*) and 1 species with male heterogamety (*A*. *officinalis*) (Fig. 3). In general, female heterogamety appears to be more frequent (10/16 species) than male heterogamety (5/16 species) in terrestrial isopods (Fig. 3 and Table 1). This does not necessarily imply that female heterogamety is the ancestral sex determination system of this clade. Indeed, the three most deeply branching species within isopods exhibit male heterogamety. Homology of the Y chromosomes between these species would constitute a strong argument for an ancestral male heterogamety in terrestrial isopods, but our approach does not allow to assess whether sex chromosomes are homologous or not. Nevertheless, sex chromosome turnovers apparently occur frequently in terrestrial isopods (see discussion below), which makes it possible that the Y chromosomes of deep-branching species are not homologous. Sequencing the genomes of *A*. *officinalis*, *H*. *brevicornis* and *A*. *aquaticus* will be useful to settle the issue of the ancestral heterogametic type in terrestrial isopods.

**Table 1:** Heterogametic types found in isopod crustaceans.

Suborder	Family	Species	Heterogametic type (Reference)	Known presence of *Wolbachia* (Reference)
Oniscidea	Armadillidiidae	*Armadillidium maculatum*	ZZ/ZW (This study)	No^27^
		*Armadillidium simoni*	Unknown	No (Unpublished)
		*Armadillidium depressum*	ZZ/ZW (This study)	Yes (This study, supplementary Table S3)
		*Armadillidium granulatum*	ZZ/ZW (This study)	Yes^28^
		*Armadillidium siculorum*	Unknown	No (This study, Supplementary Table S3)
		*Armadillidium assimile*	Unknown* (This study)	No (This study, Supplementary Table S3)
		*Armadillidium versicolor*	Unknown	No (This study, Supplementary Table S3)
		*Armadillidium vulgare*	ZZ/ZW^47^	Yes^22^
		*Armadillidium tunisiense*	Unknown	Yes^115^
		*Armadillidium nasatum*	XY/XX^46^	Yes^27^
		*Eluma purpurascens* (*caelatum*)	ZZ/ZW^37^	No^27^
	Porcellionidae	*Porcellio dilatatus petiti*	ZZ/ZW^43^	Yes^27^
		*Porcellio dilatatus dilatatus*	XY/XX^42^	Yes^116^
		*Porcellio dispar*	Unknown	Yes^27^
		*Porcellio scaber*	ZZ/ZW (This study)	Yes^27^
		*Porcellio laevis*	ZZ/ZW^48^	Yes^115^
		*Porcellionides pruinosus*	Unknown	Yes^117^
	Trachelipodidae	*Trachelipus rathkei*	ZZ/ZW^49^	Yes^28^
	Oniscidae	*Oniscus asellus*	ZZ/ZW^37^	Yes^27^
	Philosciidae	*Philoscia muscorum*	Unknown	Yes^27^
		*Chaetophiloscia elongata*	Unknown	Yes^118^
	Armadillidae	*Armadillo officinalis*	XY/XX (This study)	Yes^27^
	Tylidae	*Helleria brevicornis*	XY/XX^42^	Yes^27^
Asellota	Asellidae	*Asellus aquaticus*	XY/XX^50^	Yes^27^

(*considered as unknown because crossings of sex reversed individuals yielded inconclusive results with respect to our predictions for XY/XX and ZZ/ZW systems).

Investigation of heterogametic systems in *A*. *assimile* yielded surprising results. Indeed, all broods showed an unexpected and reproducible sex ratio bias exclusively composed of males, which cannot be readily explained by our predictions under standard male or female heterogamety (Fig. 1). We did not observe embryonic mortality in the 6 analyzed females, thus excluding a selective elimination of female embryos. Furthermore, it seems unlikely that sex reversal could affect the sex or the mortality of the progenies, especially since such an observation has never been reported for other sex reversal experiments in the literature. The coexistence of three sex chromosomes, such as Y, W and Z (as in the amphibian *Xenopus tropicalis* ^69^) or W, Y and X (as in the platyfish *Xiphophorus maculatus* ^70^) is also excluded because crossings of genetic females would necessarily produce females in the offspring whatever the combination of sex chromosomes. The hypothesis of a multi-locus sex determination system cannot be excluded, implying a combination of a major sex factor with numerous minor sex factors, as previously described in the marine isopod *Idotea balthica* ^41^. Finally, it is noteworthy that male monogeny, as observed in *A*. *assimile*, also exists in the dipteran *Mayetiola destructor* ^71^, in which sex determination is governed by the ratio between sex chromosomes and autosomes and involves maintenance/elimination of paternally derived sex chromosomes. Further experiments are needed to characterize the intriguing sex determination mechanism of *A*. *assimile*.

Our results indicate that a wide variety of sex determination systems exists in terrestrial isopods, mainly in the form of heterogametic systems. It is noteworthy that an even larger diversity of systems is known in isopods. For example, protogynous hermaphroditism was reported in *Cyathura carinata* (Cymothoida suborder)^72^ and *Gnorimosphaeroma oregonense* (Sphaeromatidea suborder)^73^, and protandric hermaphroditism in *Anilocra frontalis* (Cymothoida suborder)^74^. While these marine taxa have never been included in a phylogenetic analysis, the suborders to which they belong seemingly fall outside of the isopod clade included in this study. Cases of parthenogenetic isopods have also been reported, with coexisting forms of sexual diploid and parthenogenetic triploid forms in *Trichoniscus pusillus pusillus* ^75^. The fact that this species falls within the terrestrial isopod clade included in this study implies that the true number of transitions between sex determination mechanisms in isopods is necessarily higher than that indicated by our data. Whether these other systems are as widespread as heterogametic systems in other parts of the isopod phylogeny will be worth investigating in the future.

### Evolution of sex chromosome turnovers

We found that the distribution of sex determination mechanisms in the terrestrial isopod phylogeny requires at least three transitions between systems since the late Cretaceous (Fig. 2), but more probably between 11 and 13 according to our probabilistic analysis of reconstruction of ancestral states. Importantly, the number of transitions is necessarily underestimated, because our analysis is based on the assumption that all W chromosomes are homologous, and that all Y chromosomes are also homologous. Therefore, transitions of sex chromosomes preserving the heterogametic type cannot be taken into account in our analyses. Yet, such transitions are known to be possible in terrestrial isopods, as shown in *A*. *vulgare*. In some lines of this species, the presence of feminizing *Wolbachia* endosymbionts induced the loss of the native W sex chromosome under cytoplasmic sex determination^19, 20^, followed by the evolution of a new W sex chromosome by horizontal transfer of the *Wolbachia* genome in the *A*. *vulgare* genome^25^. This effectively led to a turnover of sex chromosomes without changing the heterogametic type.

More generally, *Wolbachia* endosymbionts are known to be widespread in terrestrial isopods^27, 28^, suggesting that these bacteria may have been involved in sex chromosome transitions in species other than *A*. *vulgare*. Our results on the distribution of sex determination systems in the terrestrial isopod phylogeny are in agreement with the prediction that if *Wolbachia* endosymbionts triggered transitions between sex determination systems, various heterogametic systems should be expected in diverse species and they should display a patchy distribution in the terrestrial isopod phylogeny. Another prediction arising from this scenario is that repeated turnovers of sex chromosomes may prevent the degeneration of sex chromosomes^10^ because of the regular relocation of sex-determining genes on different autosomal pairs. As a result, one would expect terrestrial isopods to exhibit homomorphic sex chromosomes or, at least, to show limited heteromorphy. This is congruent with the fact that homomorphic sex chromosomes are generally observed in isopods^19, 37^. However, cytogenetically homomorphic sex chromosomes could differ substantially in terms of gene content. In this context, sex reversal experiments are particularly powerful because they indicate that a simple experimental implantation of androgenic gland is sufficient to reverse an undifferentiated female into a male. This clearly implies that both male and female isopods share most if not all genes required for differentiation of either sex, and that these genes are identical in both sexes^20, 41^. The apparently labile mechanism of sex differentiation observed in terrestrial isopods probably facilitated the occurrence of feminizing endosymbionts such as *Wolbachia* ^76^. Interestingly, our approach allows us to statistically infer whether WW genotypes may be viable. Our results are consistent with largely homomorphic sex chromosomes in most ZZ/ZW species^37^. Indeed, WW genotypes are inferred to be viable in at least 7 out of 8 species and this conclusion has been experimentally confirmed in 4 of these species^37^. Furthermore, the viability of YY genotypes has been experimentally confirmed in 3 out of the four XY/XX species^42^. This is in stark contrast with the situation in mammals and insects, where YY individuals are often sterile or inviable^2, 77^.

## Conclusion

Our inference of between three and 13 transitions between heterogametic XX/XY and ZZ/ZW systems of sex determination in terrestrial isopods is quite remarkable because such transitions are believed to be rare. For example, while sex determination systems are known for thousands of insect species, only two such transitions have been inferred in these taxa, one in the ancestor of the superorder Amphiesmenoptera (Lepidoptera and Trichoptera), and one within the Tephritidae family of Diptera^78^. The paucity of such transitions may be explained by the necessary production of offspring that are homozygous for the Y or W chromosome. Such offspring may often be inviable or sterile in the context of heteromorphy, where gene content differs between sex chromosomes^2, 77, 78^. In terrestrial isopods, transitions between ZZ/ZW and XX/XY systems are likely facilitated by the fact that gene content is nearly identical between sex chromosomes, as indicated by the viability of WW individuals in most ZZ/ZW isopod species. Overall, the repeated and likely recent transitions of heterogametic systems in terrestrial isopods are consistent with the hypothesis that *Wolbachia* endosymbionts have had a strong impact on the evolution of sex determination mechanisms in these taxa. Terrestrial isopods thus provide a great opportunity to further understand the evolutionary forces driving transitions and early evolution of sex chromosomes.

The resulting turnovers also raise the question of the role of *Wolbachia* in host divergence and speciation. Sex chromosomes can play key roles in phenotypic evolution and speciation. For example, studies based on genetic mapping have shown that hybrid deficiencies, such as hybrid sterility, mostly map to sex chromosomes^79, 80^. Sex chromosomes often harbor genes linked to mate choice or behavioral isolation in a variety of species such as Poeciliid fishes^81^, flycatchers^82^, stickleback^83^ or finches^84^. Whether such sexually antagonistic genes have sufficient time to accumulate or to be expressed in a sex-biased fashion on otherwise homomorphic sex chromosomes in terrestrial isopods is an open question deserving further investigation. More generally, it will be interesting to assess the contribution that feminizing *Wolbachia* may have had as a trigger of sex chromosome turnovers in relation to speciation, in generating the large, worldwide diversity of terrestrial isopods^55, 85^.

## Materials and Methods

### RNA preparation, sequencing and *de novo* assembly

Transcriptome data were generated for 19 species of terrestrial isopods (Supplementary Table S1) available in our laboratory. Total RNA was extracted from one or two adult individuals frozen in liquid nitrogen and grinded with a mortar and pestle. The resulting powders were processed using the RNeasy extraction protocol (Qiagen). Quality and quantity of total RNA was examined using a MCE-202 MultiNA (Shimadzu) and a Qubit 2.0 (Invitrogen). For each species, 1–12 µg of total RNA were extracted at concentrations ranging from 15.5 ng.µL^−1^ to 240 ng.µL^−1^. Paired-end sequencing libraries were constructed and sequenced by Eurofins (Germany) on one multiplexed lane of an Illumina HiSeq2500 platform, with a read length of 125 bp. The total number of reads obtained ranged from 12,766,990 to 24,948,176 depending on the species (Supplementary Table S1). For *T*. *rathkei*, RNA was isolated from head, leg, and gonad tissues from one male and one female, both wild-caught at Rice Creek Field Station in Oswego, NY, using an SV Total RNA Isolation Kit (Promega). Libraries for each sample were prepared at the State University of New York at Buffalo Genomics Core Facility, barcoded, pooled, and sequenced in a single paired-end Illumina HiSeq2500 lane with a read length of 100 bp. Sequence data of the 19 transcriptomes generated in this study have been deposited in GenBank under accession numbers SRX2600476-SRX2600493 and SRR5198726-SRR5198727. Transcriptome data from five additional crustacean species were included in our analyses: *A*. *vulgare* ^86^, *A*. *nasatum* ^86^, *A*. *aquaticus* ^87^, *C*. *quadricarinatus* ^88^ and *T*. *saltator* ^89^ (all SRA accession numbers are provided in Supplementary Table S1).

Read quality was analyzed with FastQC (version 0.11.4, http://www.bioinformatics.babraham.ac.uk/projects/fastqc). Removal of low quality reads and sequencing adaptors was performed with Trimmomatic (version 0.33^90^), setting the ILLUMINACLIP palindrome clip threshold at 30 and the simple clip threshold at 10. The quality threshold was set to a minimum Phred score of 20. Filtered reads were assembled using the Trinity de novo Assembler (release 2014 07 17^91^). Assembly metrics for each transcriptome are provided in Supplementary Table S6.

### Marker selection and phylogenetic inferences

To establish a set of orthologous genes to be used in phylogenetic analyses, we first translated each transcriptome using Virtual Ribosome (version 1.1^92^). The first translation strategy consisted in keeping the longest contigs starting with a start codon among the 6 possible open reading frames (ORFs) (parameters used: -readingframe = all, -orf = any). The second strategy consisted in keeping the longest contigs ending with a stop codon among the 6 possible ORFs (parameters used: -readingframe = all, -orf = none). Of these two datasets of translated expected proteins, the longest sequences were kept (including sequences that had both a start and a stop codon, or sequences that had only a start or a stop codon).

Orthology relationships among all terrestrial isopod proteomes (i.e. all species except *T*. *saltator*, *C*. *quadricarinatus* and *A*. *aquaticus*) were inferred using the OrthoMCL pipeline^93^. Among the 45,724 orthology groups returned, 2,168 contained at least 20 of the 21 taxa. We focused on orthogroups containing at most 3 paralogous sequences, corresponding to 90 orthogroups. Annotation of these orthogroups was performed using BlastX^94^ against the non-redundant (NR) database of NCBI (version January 2016) (Supplementary Table S7). Two orthogroups showing no significant hit to any known protein were removed from this dataset, as they could not be confidently assigned to a terrestrial isopod origin (orthogroups 39 and 60). The orthologous sequences from *T*. *saltator*, *C*. *quadricarinatus* and *A*. *aquaticus* were identified using a one-to-one reciprocal best BlastP alignment approach on the 88 terrestrial isopod orthogroups. This search was performed after the definition of the terrestrial isopod orthogroups to avoid underestimating the amount of orthogroups determined by OrthoMCL due to the large genetic distances between terrestrial isopods and *T*. *saltator* and *C*. *quadricarinatus*, or because of a lack of data for *A*. *aquaticus*, which transcriptome contained 3 times fewer assembled sequences and 7 times fewer nucleotides than other transcriptomes (Supplementary Table S6). All 88 orthologous sequences were found in *T*. *saltator* and *C*. *quadricarinatus* and 66 orthologous sequences were found *A*. *aquaticus*.

All sequences within each orthogroup were codon-aligned using default parameters in Geneious (version 7.0.6^95^). Each multiple alignment was then trimmed with GBLOCKS (version 0.91b^96^) to remove ambiguous regions. All alignments were finally concatenated into a single nexus interleaved file using SequenceMatrix (version 1.8^97^). To determine the best-fit model of nucleotide substitution, we used Jmodeltest (version 2.1.7^98^). The best substitution model was the GTR + G + I, according to all information criteria. Maximum-likelihood analyses were performed on the concatenated codon alignment using RAxML (version 7.4.6^99^) with 100 independent replicates followed by 200 replicates of bootstrap resampling. Bootstrap values were subsequently mapped onto the optimal consensus tree obtained from the 100 independent searches. The two non-isopod species *T*. *saltator* (Amphipoda) and *C*. *quadricarinatus* (Decapoda) were used as outgroups.

To include two additional species for which the heterogametic system is known (*P*. *d*. *dilatatus* [XY/XX] and *P*. *d*. *petiti* [ZZ/ZW]), we selected ten markers (5 nuclear and 5 mitochondrial) on the basis of their length and phylogenetic signal and we Sanger-sequenced them. PCRs were performed using degenerate primers as follows: 3 min at 94 °C for the initial denaturing step, followed by 35 cycles of 30 s at 94 °C; 30 s at 54 °C, 56 °C, 57 °C or 58 °C (depending on the melting temperature of the primers) and 1 min at 72 °C. The final elongation step was 10 min at 72 °C. Purified PCR products were sequenced on an ABI PRISM 3130xl automated sequencer (Applied Biosystems). Primer sequences, PCR product sizes and associated melting temperatures are listed in Supplementary Table S8.

### Divergence time analyses

Divergence time analyses were performed using BEAST (version 1.8.3^100^) on the combined alignment including 88 markers for all species except *P*. *d*. *dilatatus* and *P*. *d*. *petiti* (10 markers) and *A*. *aquaticus* (66 markers). We used the RAxML topology tree as starting tree and conducted a random clock analysis. To obtain the Bayesian posterior probability for each node, the same analysis was performed with no constrained topology as starting tree. The rate parameter (*clock.rate*) was left to default prior distribution (CTMC Rate Reference with an initial value of 0.01^101^). A Poisson distribution was used for the number of local clocks (*rateChanges* parameter, mean 0.693) and an exponential prior distribution was set for the relative rates among the random local clock (*localClock.relativeRates*, mean 1.0, initial value 0.001). Base frequencies (*frequencies*) and proportion of invariant sites parameter (*pInv*) were left to their initial priors (uniform distribution with an initial value of 0.25, and uniform distribution with an initial value of 0.5, respectively). The gamma shape parameter (*alpha*) was also left to his initial prior (exponential distribution, initial value 0.5, mean 0.5). Three fossil calibration points were used to calibrate our molecular clock analysis. A first fossil found in amber from the early beginning of the Oligocene in Germany provides a minimum age of 23.03 million years (My) for the *Armadillidium* genus^102^. A second fossil specimen in amber from the Oligocene close to the Baltic Sea in Northern Europe indicates a minimum age of 33.9 My for the *Porcellio* genus^103^. Third, the earliest terrestrial isopod fossils were found in amber from the Lower Cretaceous of Northern Spain, which allowed us to set the minimum age of terrestrial isopods (Oniscidea) at 110 My^104, 105^. All calibrations were modeled following a gamma prior distribution (shape 2.0), with an offset value fixed at the oldest age bordering the time interval containing the related fossil (*Armadillidium*: Miocene 5.333 to 23.03 My; *Porcellio*: Oligocene 23.03 to 33.9 My).

Markov chain Monte Carlo (MCMC) were run for 50 million generations, and parameter values were sampled every 1,000 generations. The log file output was processed with LogCombiner (version 1.8.3, implemented in BEAST) with 10% burn-ins removed. The resulting log file was analyzed with Tracer (version 1.6, also implemented in BEAST) to confirm that the MCMC converged with an effective sample size greater than 200 for all priors. The maximum clade credibility tree was created with TreeAnnotator (part of BEAST suit), and the resulting tree was visualized with FigTree (version 1.4.2, http://tree.bio.ed.ac.uk/software/figtree) and R software (version 3.1.1^106^), using the packages *strap* ^107^ and *phyloch* ^108^.

### Substitution rates

Using the RAxML topology tree and a nuclear codon alignment of the 81 concatenated nuclear sequences, maximum likelihood estimates of branch-specific synonymous and non-synonymous substitution rates (dS and dN) were calculated with CodeML, a program from the PAML software package (version 4.9c^109, 110^), with default parameters. The analysis was also conducted independently with a concatenated codon alignment of the 7 mitochondrial markers. To scale the substitution rate per site to absolute substitution rate, dS values calculated with CodeML were divided by the BEAST median estimate of branch age for each branch of the tree, in both nuclear and mitochondrial analyses. The global dS of Oniscidea was calculated by adding dS values of all branches, divided by the sum of ages calculated for all branches of the tree, for both nuclear and mitochondrial sequences.

### Sex-reversal experiments

**Figure 4:**
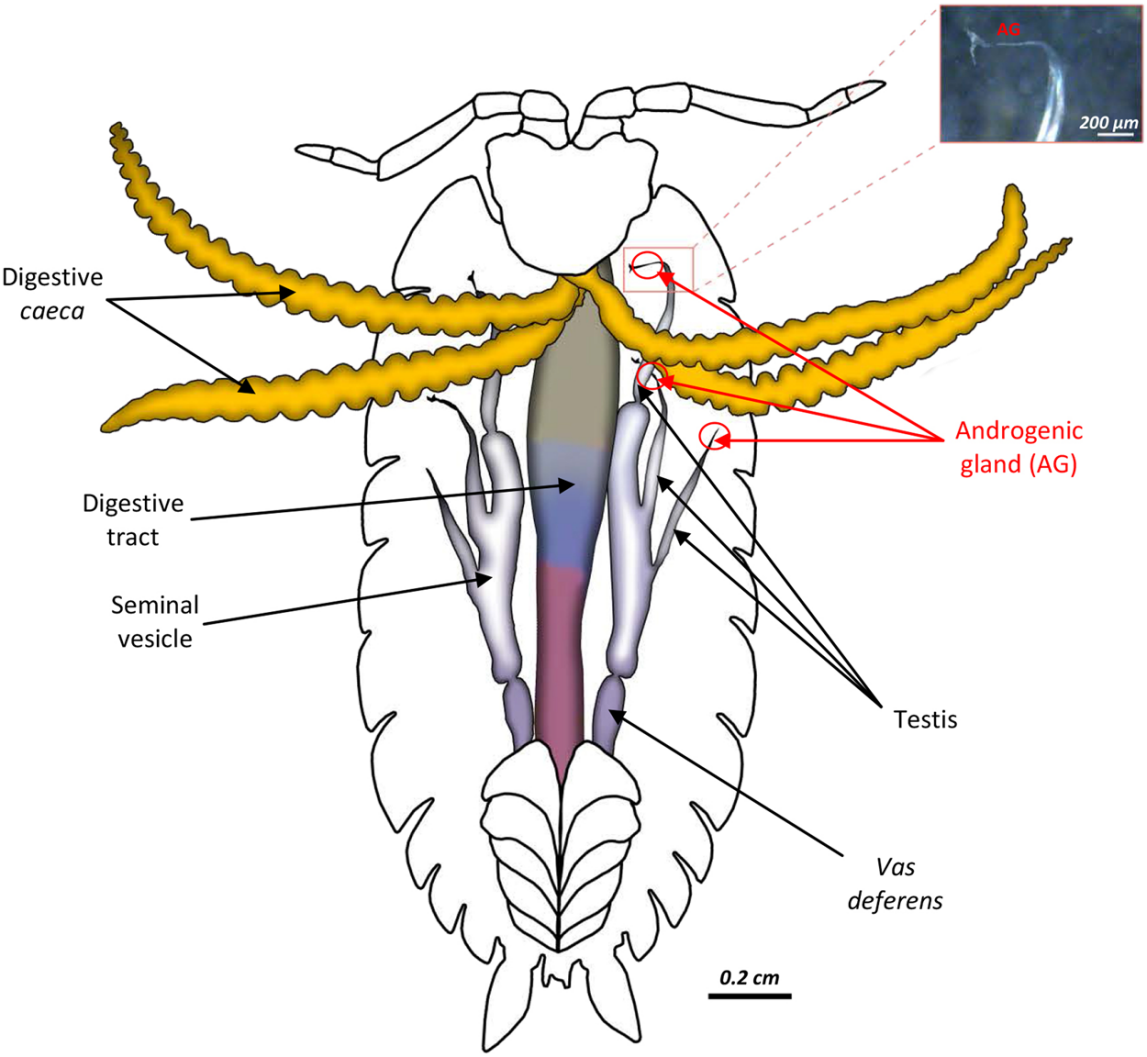
Schematics of the internal organs of a terrestrial isopod (*Oniscus asellus*) in ventral view. Androgenic glands, composed of a cellular mass excreting androgenic hormone, are located at the top of each testis, included in the suspensory filament. Depending on the species, its size is 250–700 µm in length and 20–150 µm in width.

Sex reversal experiments were attempted on 15 terrestrial isopod species listed in Supplementary Table S2. Sex reversion of genetic females into functional phenotypic males (neomales) was performed by injection of androgenic glands into female embryos following the technique used in older study^42^. In crustaceans, androgenic glands are located at the end of each male testes (Fig. 4). They control androgenic hormone production, which itself triggers male sexual differentiation^40^. For each species, fresh androgenic glands were first dissected under a magnifying binocular from three adult males placed in Ringer solution (NaCl: 394 mM, KCl: 2 mM, CaCl_2_: 2 mM, NaHCO_3_: 2 mM). Androgenic glands were collected and stored in Ringer solution. Then, they were injected through a hole pierced in the tergite of the 6^th^ thoracic segment of juvenile females. From 1 to 3 androgenic glands were implanted in each developing female. Injected genetic females were between the 4^th^ and 6^th^ molt stage after birth, which corresponds to the molt stages during which sex differentiation takes place in *A*. *vulgare* ^38^. These young females were selected for injections by comparison with male siblings of the same size (between 3 and 5 mm in length depending on the species) which were at the beginning of sexual differentiation. Early differentiating males can morphologically be distinguished from early differentiating females by the development of two ventral copulating pleopods.

### Genetic crosses and statistical analyses of F1 progenies

After full sexual differentiation (20–28 weeks post-injection), neomales were crossed with their sisters (genetic females) to produce F1 progenies. Analyses of the sex-ratios of these progenies allowed us to deduce the type of heterogametic system in the various species. In the case of a male heterogametic system (XY/XX), neomales (i.e. sex-reversed genetic females) are XX and crossing them with their XX sisters is expected to produce progenies comprising 100% XX females (Fig. 1). In the case of a female heterogametic system (ZZ/ZW), neomales are ZW and crossing them with their ZW sisters is expected to produce progenies comprising 25% ZZ males, 25% WW females and 50% ZW females. Thus, we predict a proportion of 3/4 females if the WW genotype is viable, and 2/3 females if the WW genotype is not viable (Fig. 1).

To rule out any confounding effect due to feminizing *Wolbachia* infection (which would also result in female-biased sex ratios), total DNA was extracted from the head of all mothers after they produced their progenies, using the Qiagen DNeasy Blood and Tissue kit according to the manufacturer’s instructions. Success of DNA extractions was checked by PCR amplification of the mitochondrial cytochrome oxidase I marker^111^. Next, absence of *Wolbachia* infection was checked by PCR using *Wolbachia*-specific primers for the wsp and ftsZ markers^112, 113^.

Sex ratios of F1 progenies were analyzed using χ² tests. First, we assessed whether sex ratios were statistically different from a balanced sex ratio (1:1). If not, we concluded that the progeny originated from a cross between a genetic male (instead of a neomale) and a genetic female, implying that androgenic glands had been injected into an undifferentiated genetic male instead of a female during the sex reversal experiment. We also performed χ² tests to assess whether observed sex ratios were significantly different from 1/3♂:2/3♀, in which case the WW genotype would be deemed viable, or from 1/4♂:3/4♀, in which case the WW genotype would be deemed inviable. These statistical analyses were performed with the R software (version 3.1.1^106^). The significance level was set at 0.05.

### Identification of sex chromosome transitions

In the reconstruction of ancestral traits, we assumed all XY/XX chromosomes are homologous and all ZZ/ZW chromosomes are homologous. This constitutes a conservative hypothesis, limiting our estimates to a minimal number of transitions between heterogametic systems. To consider the possibility of a different and undetermined sex determination system in *A*. *assimile*, two kinds of discrete states were defined to conduct ancestral states analysis: (1) three discrete states were defined according to sex chromosome types: male heterogamety (XY/XX), female heterogamety (ZZ/ZW) and undetermined (in the case of *A*. *assimile*), and (2) only two discrete states were defined (XY/XX or ZZ/ZW) and *A*. *assimile* was treated as the other species for which no information on sex determination system is available. The evolution of character states was inferred using the Parsimony Ancestral States reconstruction of the MESQUITE software (version 3.04, build 725^51^). This reconstruction infers the most parsimonious (i.e. the lowest) number of possible transitions to explain the distribution of sex determination systems across the phylogeny. In addition, maximum likelihood-based ancestral state reconstructions analyses were performed using the R *Phytools* package^114^ to take into account possibly unobserved transitions between heterogametic systems. In both defined conditions of discrete states (1) and (2), equal probability between sex determination systems (XY/XX, ZZ/ZW and *A*. *assimile* system in (1)) was set for species with unknown sex determination mechanisms. Outgroup species were excluded from the reconstructions. Analyses were performed using the one-parameter equal rates model (“ER” model) and the symmetric model in which forward and reverse transitions between states are constrained to be equal (“SYM” model). All maximum likelihood simulations were conducted with 1,000 stochastic mappings.

## Electronic supplementary material

Supplementary Material
