# Author Correction: Diversity and evolution of sex determination systems in terrestrial isopods

**DOI:** 10.1038/s41598-018-25423-7

**Published:** 2018-04-27

**Authors:** Thomas Becking, Isabelle Giraud, Maryline Raimond, Bouziane Moumen, Christopher Chandler, Richard Cordaux, Clément Gilbert

**Affiliations:** 1 Université de Poitiers, UMR CNRS 7267 Ecologie et Biologie des Interactions, Equipe Ecologie Evolution Symbiose, TSA 51106, 86073 Poitiers Cedex 9, France; 2 Department of Biological Sciences, SUNY Oswego, Oswego, New York 13126 USA

Correction to: *Scientific Reports* 10.1038/s41598-017-01195-4, published online 24 April 2017

This Article contains errors where seven genetic markers were mistakenly attributed to the mitochondrial genome rather than the nuclear genome, like the other 81 markers.

As a result, in the Results section, under subheading ‘Phylogenetic analyses’,

“To reconstruct the phylogeny of terrestrial isopods, we generated transcriptome data for 19 species available in our laboratory and used the transcriptomes of 5 other relevant crustaceans that were publicly available (Supplementary Table S1). Our search for orthologous genes in the transcriptomes of 24 crustacean species yielded 88 markers (81 nuclear and 7 mitochondrial). Out of 22 isopod species, we found 76–88 markers in all species but *Helleria brevicornis* (67 markers) and *Asellus aquaticus* (66 markers). In addition, all 88 markers were found in the two outgroup species *Cherax quadricarinatus* (Australian freshwater crayfish) and *Talitrus saltator* (European sand hopper). When combined, these markers produced a 69,570 bp-long alignment with a very low amount of missing data (7%). Two subspecies for which the heterogametic system is known were not included in our RNA-seq experiment: *Porcellio dilatatus dilatatus* (XY/XX)^42^ and *Porcellio dilatatus petiti* (ZZ/ZW)^43^. To include these species in our phylogeny at lower cost, we first performed phylogenetic analyses of each of the 88 markers independently. Among the 35 markers that independently produced a topology identical to the one retrieved with the combined alignment, we selected the 10 longest ones (5 nuclear and 5 mitochondrial) and Sanger-sequenced them in the two *P*. *dilatatus* subspecies. The 10 markers sequenced in *P*. *d*. *dilatatus* (total length: 3,054 bp) and *P*. *d*. *petiti* (total length: 3,284 bp) were analyzed both separately as a combined 10-marker alignment and together with the other 78 markers. The phylogenetic analysis based on the 88-marker alignment produced a fully resolved tree, with all nodes supported by bootstrap values of 100% and all Bayesian posterior probabilities equal to 1 (Fig. 2, Supplementary Figure S1). The 10-marker tree was fully congruent with the 88-marker tree and strongly supported as well (all bootstrap values but one ≥95%, Supplementary Figure S1). We also compared the topology of the tree obtained using a combined alignment of the mitochondrial markers only (7 out of 88; alignment length: 5,847 bp) to that obtained with a combined alignment of the nuclear markers only (81 out of 88; alignment length: 63,723 bp). The two topologies were identical with each other and with the 88-marker and 10-marker trees (10 out of 88; alignment length: 10,773 bp), and they were both strongly supported (bootstrap scores for mitochondrial markers: all but two ≥95%; for nuclear markers: all but one ≥95%, Supplementary Figure S1).”

should read:

“To reconstruct the phylogeny of terrestrial isopods, we generated transcriptome data for 19 species available in our laboratory and used the transcriptomes of 5 other relevant crustaceans that were publicly available (Supplementary Table S1). Our search for orthologous genes in the transcriptomes of 24 crustacean species yielded 88 nuclear markers. Out of 22 isopod species, we found 76–88 markers in all species but *Helleria brevicornis* (67 markers) and *Asellus aquaticus* (66 markers). In addition, all 88 markers were found in the two outgroup species *Cherax quadricarinatus* (Australian freshwater crayfish) and *Talitrus saltator* (European sand hopper). When combined, these markers produced a 69,570 bp-long alignment with a very low amount of missing data (7%). Two subspecies for which the heterogametic system is known were not included in our RNA-seq experiment: *Porcellio dilatatus dilatatus* (XY/XX)^42^ and *Porcellio dilatatus petiti* (ZZ/ZW)^43^. To include these species in our phylogeny at lower cost, we first performed phylogenetic analyses of each of the 88 markers independently. Among the 35 markers that independently produced a topology identical to the one retrieved with the combined alignment, we selected the 10 longest ones and Sanger-sequenced them in the two *P*. *dilatatus* subspecies. The 10 markers sequenced in *P*. *d*. *dilatatus* (total length: 3,054 bp) and *P*. *d*. *petiti* (total length: 3,284 bp) were analyzed both separately as a combined 10-marker alignment and together with the other 78 markers. The phylogenetic analysis based on the 88-marker alignment produced a fully resolved tree, with all nodes supported by bootstrap values of 100% and all Bayesian posterior probabilities equal to 1 (Fig. 2, Supplementary Figure S1). The 10-marker tree was fully congruent with the 88-marker tree and strongly supported as well (all bootstrap values but one ≥95%, Supplementary Figure S1).”

In the same section, under subheading ‘Substitution rates’,

“Mitochondrial and nuclear codon alignments were used to estimate the rate of silent substitutions across isopods. When scaled to the fossil-calibrated chronogram, substitution rates in isopods were estimated at 3.89 × 10^−9^ and 4.17 × 10^−9^ silent substitutions per year for mitochondrial and nuclear sequences, respectively (Supplementary Figure S3). We also calculated the rate of non-synonymous/synonymous substitutions (dN/dS) for the two datasets. Both mitochondrial and nuclear dN/dS ratios were <1, thus indicating that our markers evolve under strong purifying selection (mitochondrial dN/dS = 0.28; nuclear dN/dS = 0.07).”

should read:

“Codon alignment was used to estimate the rate of silent substitutions across isopods. When scaled to the fossil-calibrated chronogram, substitution rate in isopod was estimated at 4.17 × 10^−9^ silent substitutions per year (Supplementary Figure S3). We also calculated the rate of non-synonymous/synonymous substitutions. The dN/dS ratio was <1, thus indicating that our markers evolve under strong purifying selection (dN/dS = 0.07).”

In the Discussion section, under subheading ‘Phylogenetic analyses’,

“In this study, we used a large combination of 88 nuclear and mitochondrial molecular markers obtained by transcriptome sequencing to establish a fully resolved molecular phylogeny of terrestrial isopods (Supplementary Figure S1).”

should read:

“In this study, we used a large combination of 88 nuclear molecular markers obtained by transcriptome sequencing to establish a fully resolved molecular phylogeny of terrestrial isopods (Supplementary Figure S1).”

In the same section, under subheading ‘Divergence times and estimates of substitution rates’,

“Unlike insects, terrestrial isopods do not possess a waxy epicuticle, thus complicating the fossilization process^57^. The terrestrial isopod fossil record is thus relatively poor, implying that the divergence times of these taxa must be interpreted with caution. Our results differ from those obtained in an earlier study^58^, which included several terrestrial isopods as part of an investigation of the evolution of deep sea isopods. This discrepancy is likely due to the fact that they did not calibrate any node within terrestrial isopods^58^. Thus, our analyses are more likely to be closer to the true divergence times for this clade.

Our study also allowed us to calculate absolute silent substitution rates (dS) in terrestrial isopods, which are very similar between mitochondrial and nuclear sequences (3.89 × 10^−9^ and 4.17 × 10^−9^ silent substitutions per years, respectively). In comparison, several studies based on mitochondrial COI sequences from crustacean species showed substantial dS variations depending on the studied order. Whereas mitochondrial dS rates similar to those reported here were found in copepods (9.3 × 10^−9^, *Tigriopus* genus^59^) and aquatic isopods (4.6 × 10^−9^, *Stenasellus* genus^60^), it has been established that this rate might be 20 times higher in decapods (86 × 10^−9^ in genus *Alpheus*^61^) for the same sequence (COI). More surprisingly, mitochondrial genes usually evolve at an elevated rate compared to nuclear genes, e.g. 11 to 62-fold higher in mammals and 1.4 to 18-fold higher in insects^62, 63^. By contrast, we did not observe such a variation in our datasets where mitochondrial and nuclear dS appear to be similar. To some extent, such a situation has already been reported in corals^64^, fungi^65^ and sponges^66^. In these taxa, a specific mitochondrial DNA repair function could be responsible for the low levels of mitochondrial divergence. In spite of the availability of well assembled mitochondrial genomes, no such gene is known in terrestrial isopods^67, 68^ and at present, we cannot explain why mitochondrial and nuclear dS are similar.”

should read:

“Unlike insects, terrestrial isopods do not possess a waxy epicuticle, thus complicating the fossilization process^57^. The terrestrial isopod fossil record is thus relatively poor, implying that the divergence times of these taxa must be interpreted with caution. Our results differ from those obtained in an earlier study^58^, which included several terrestrial isopods as part of an investigation of the evolution of deep sea isopods. This discrepancy is likely due to the fact that they did not calibrate any node within terrestrial isopods^58^. Thus, our analyses are more likely to be closer to the true divergence times for this clade.”

In the Methods section, under subheading ‘Marker selection and phylogenetic inferences’,

“To include two additional species for which the heterogametic system is known (*P*. *d*. *dilatatus* [XY/XX] and *P*. *d*. *petiti* [ZZ/ZW]), we selected ten markers (5 nuclear and 5 mitochondrial) on the basis of their length and phylogenetic signal and we Sanger-sequenced them.”

should read:

“To include two additional species for which the heterogametic system is known (*P*. *d*. *dilatatus* [XY/XX] and *P*. *d*. *petiti* [ZZ/ZW]), we selected ten markers on the basis of their length and phylogenetic signal and we Sanger-sequenced them.”

In the same section, under subheading ‘Substitution rates’,

“Using the RAxML topology tree and a nuclear codon alignment of the 81 concatenated nuclear sequences, maximum likelihood estimates of branch-specific synonymous and non-synonymous substitution rates (dS and dN) were calculated with CodeML, a program from the PAML software package (version 4.9c^109, 110^), with default parameters. The analysis was also conducted independently with a concatenated codon alignment of the 7 mitochondrial markers. To scale the substitution rate per site to absolute substitution rate, dS values calculated with CodeML were divided by the BEAST median estimate of branch age for each branch of the tree, in both nuclear and mitochondrial analyses. The global dS of Oniscidea was calculated by adding dS values of all branches, divided by the sum of ages calculated for all branches of the tree, for both nuclear and mitochondrial sequences.”

should read:

“Using the RAxML topology tree and a nuclear codon alignment of the 81 concatenated nuclear sequences, maximum likelihood estimates of branch-specific synonymous and non-synonymous substitution rates (dS and dN) were calculated with CodeML, a program from the PAML software package (version 4.9c^109, 110^), with default parameters. To scale the substitution rate per site to absolute substitution rate, dS values calculated with CodeML were divided by the BEAST median estimate of branch age for each branch of the tree. The global dS of Oniscidea was calculated by adding dS values of all branches, divided by the sum of ages calculated for all branches of the tree.”

In the Supplementary Information file, the values indicating the bootstrap values of both nuclear and mitochondrial markers should be removed from Figure S1. The remaining written values next to each node correspond to the Bayesian posterior probabilities from BEAST analysis and to the bootstrap score from the Maximum Likelihood analysis using all 88 markers and the 10 PCR markers. The corrected Figure S1 and its accompanying legend appear below as Figure 1.

**Figure 1:**
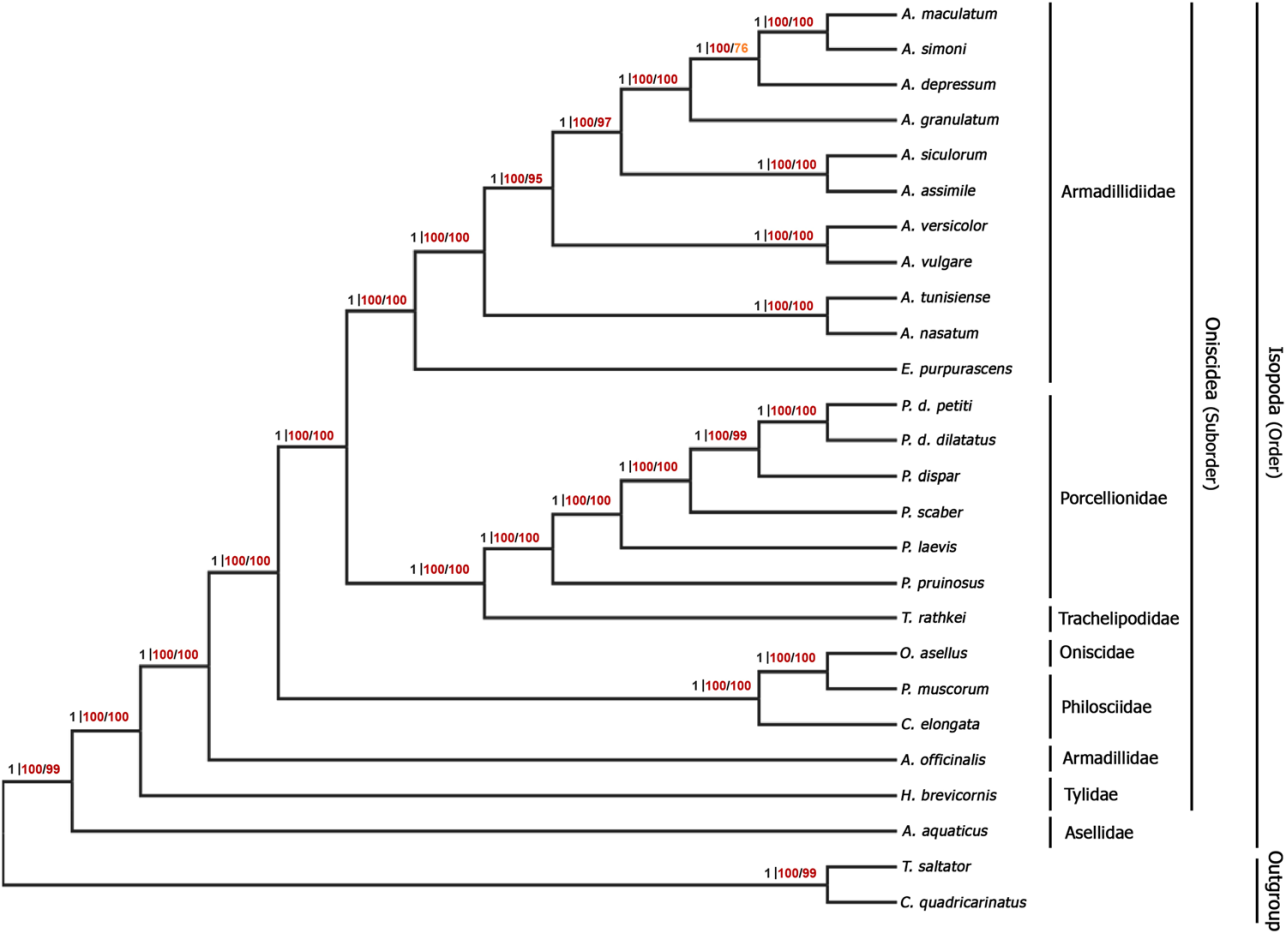
Cladogram showing the relationships among the 26 species used in this study. The selected model is GTR + I + G for each combination of markers. The values written next to each node correspond to the Bayesian posterior probabilities from BEAST analysis (without a constrained topology) and to the bootstrap score from the Maximum Likelihood analysis (200 bootstrap replicates) using all 88 markers (alignment length : 69,570 bp) / PCR markers (10 markers, alignment length : 10,773 bp), following this order.

In addition, only the silent substitution rate (dS) of each branch of isopods (blue values) should be indicated in Figure S3. The corrected Figure S3 and its accompanying legend appear below as Figure 2.

**Figure 2:**
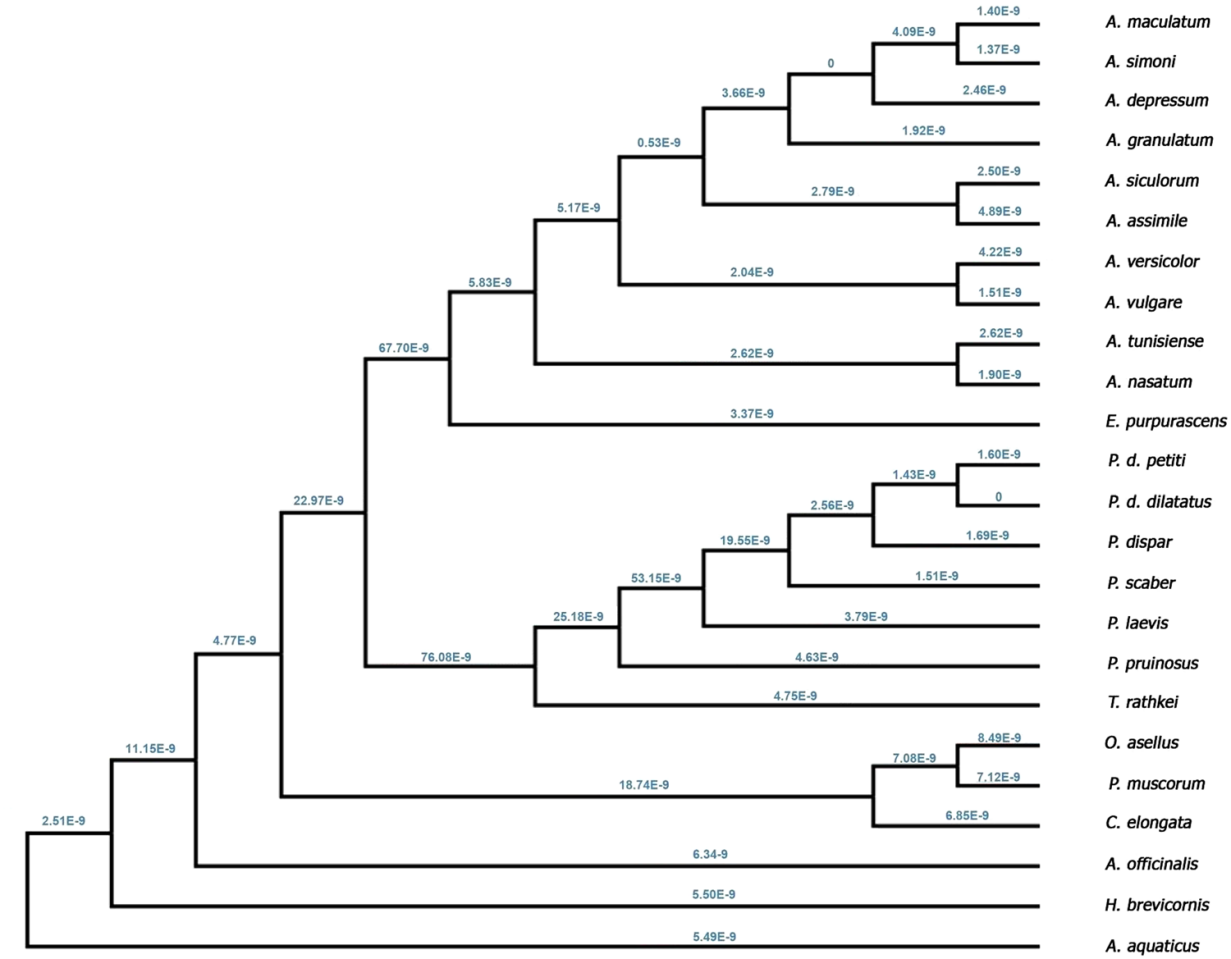
Cladogram indicating the silent substitution rate (dS) of each branch of isopods (blue). The per-site rate of amino acid replacements/ silent substitution rate ratio (dN/dS) is estimated at 0.0674.

