# Supplementary material for: Diversity and evolution of sex determination systems in terrestrial isopods
Source: Sci Rep. 2017 Apr 24;7:1084. doi: 10.1038/s41598-017-01195-4 (PMC5430756; doi:10.1038/s41598-017-01195-4)
Supplement: Supplementary file 1 — Supplementary Material [file 41598_2017_1195_MOESM1_ESM.pdf]

Diversity and evolution of sex determination systems in terrestrial isopods

Thomas Becking[1], Isabelle Giraud[1], Maryline Raimond[1], Bouziane Moumen[1], Christopher Chandler[2], Richard Cordaux[1,#,*] and Clément Gilbert[1,#,*]

[1]Université de Poitiers, UMR CNRS 7267 Ecologie et Biologie des Interactions, Equipe Ecologie Evolution Symbiose, TSA 51106, 86073 Poitiers Cedex 9, France

[2]Department of Biological Sciences, SUNY Oswego, Oswego, New York 13126

# These authors contributed equally to this work

* Corresponding authors: RC (richard.cordaux@univ-poitiers.fr) and CG (clement.gilbert@univ-poitiers.fr)

Supplementary Figure legends

Supplementary Figure S1. Cladogram showing the relationships among the 26 species used in this study. The selected model is GTR + I + G for each combination of markers. The values written next to each node correspond to the Bayesian posterior probabilities from BEAST analysis (without a constrained topology) and to the bootstrap score from the Maximum Likelihood analysis (200 bootstrap replicates) using all 88 markers (alignment length : 69,570 bp) / nuclear markers (81 markers, alignment length : 63,723 bp) / mitochondrial markers (7 markers, alignment length : 5,847 bp) / PCR markers (10 markers, alignment length : 10,773 bp), following this order.

Supplementary Figure S2 : Chronogram of terrestrial isopod crustaceans, obtained using BEAST and the tree shown in Supplementary Figure S1 as a backbone. Branch length is scaled to time, with nodal ages corresponding to median posterior estimates. Grey bars represent the Highest Posterior Density (HPD) intervals at 95%. Orange bars denote the calibration points.

Supplementary Figure S3. Cladogram indicating the silent substitution rate (dS) of each branch of isopods for mitochondrial sequences (red), nuclear sequences (blue) and the ratio between mitochondrial dS and nuclear dS (black). The per-site rate of amino acid replacements/ silent substitution rate ratio (dN/dS) of mitochondrial and nuclear dataset are estimated respectively at 0.2785 and 0.0674.

Supplementary Table legends

Supplementary Table S1. Information on transcriptomic data used.

Supplementary Table S2. Detailed information on the sex reversal experiment success.

Supplementary Table S3. Statistical analyses of sex ratio from the broods obtained in crosses between females and neomales. Red cells indicate females infected by *Wolbachia* (excluded results). Orange cells indicate that the progeny sex-ratio is not significantly different from 50/50 (excluded results). Green cells indicate that the progeny sex-ratio is significantly different from 50/50 (included results).

Supplementary Table S4. Summary of the statistics used to determine the heterogametic systems.

Supplementary Table S5. Ancestral states reconstruction of the estimated number of transitions between sex determination systems. The numbers written for the maximum likelihood analysis correspond to the mean number of transitions estimated with 1,000 stochastic mappings (± standard deviation).

Supplementary Table S6. Metrics of the 19 isopod transcriptomes assembled in this study.

Supplementary Table S7. Detailed annotation of the 88 markers used in this study (5 first best BLASTX versus NCBI non-redundant database hit are shown).

Supplementary Table S8. Sequences, PCR product size, and melting temperature of the primers designed to complete the phylogeny.

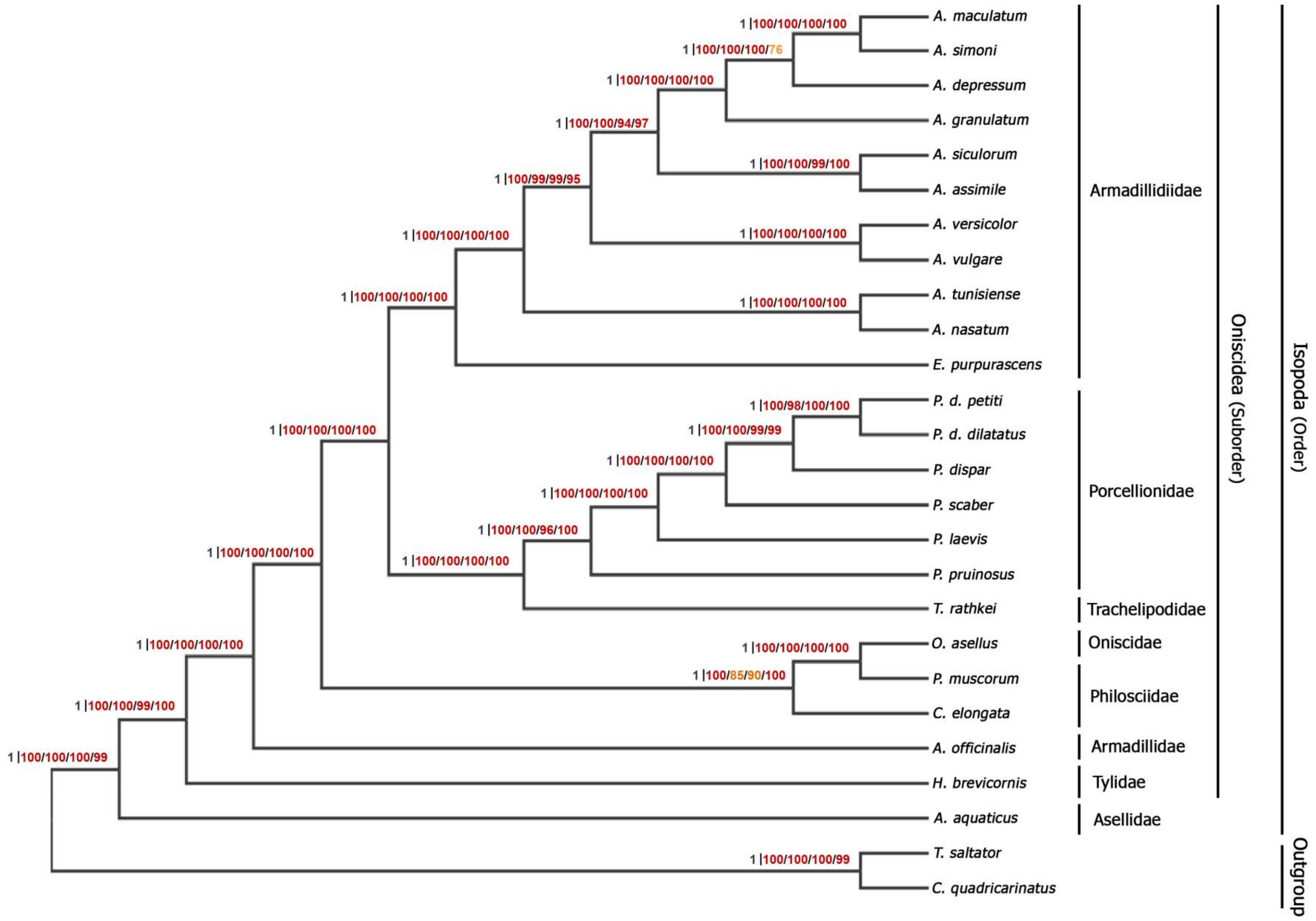

Figure S1. Cladogram showing the relationships among the 26 species used in this study. The selected model is GTR + I + G for each combination of markers. The values written next to each node correspond to the Bayesian posterior probabilities from BEAST analysis (without a constrained topology) and to the bootstrap score from the Maximum Likelihood analysis (200 bootstrap replicates) using all 88 markers (alignment length : 69,570 bp) / nuclear markers (81 markers, alignment length : 63,723 bp) / mitochondrial markers (7 markers, alignment length : 5,847 bp) / PCR markers (10 markers, alignment length : 10,773 bp), following this order.

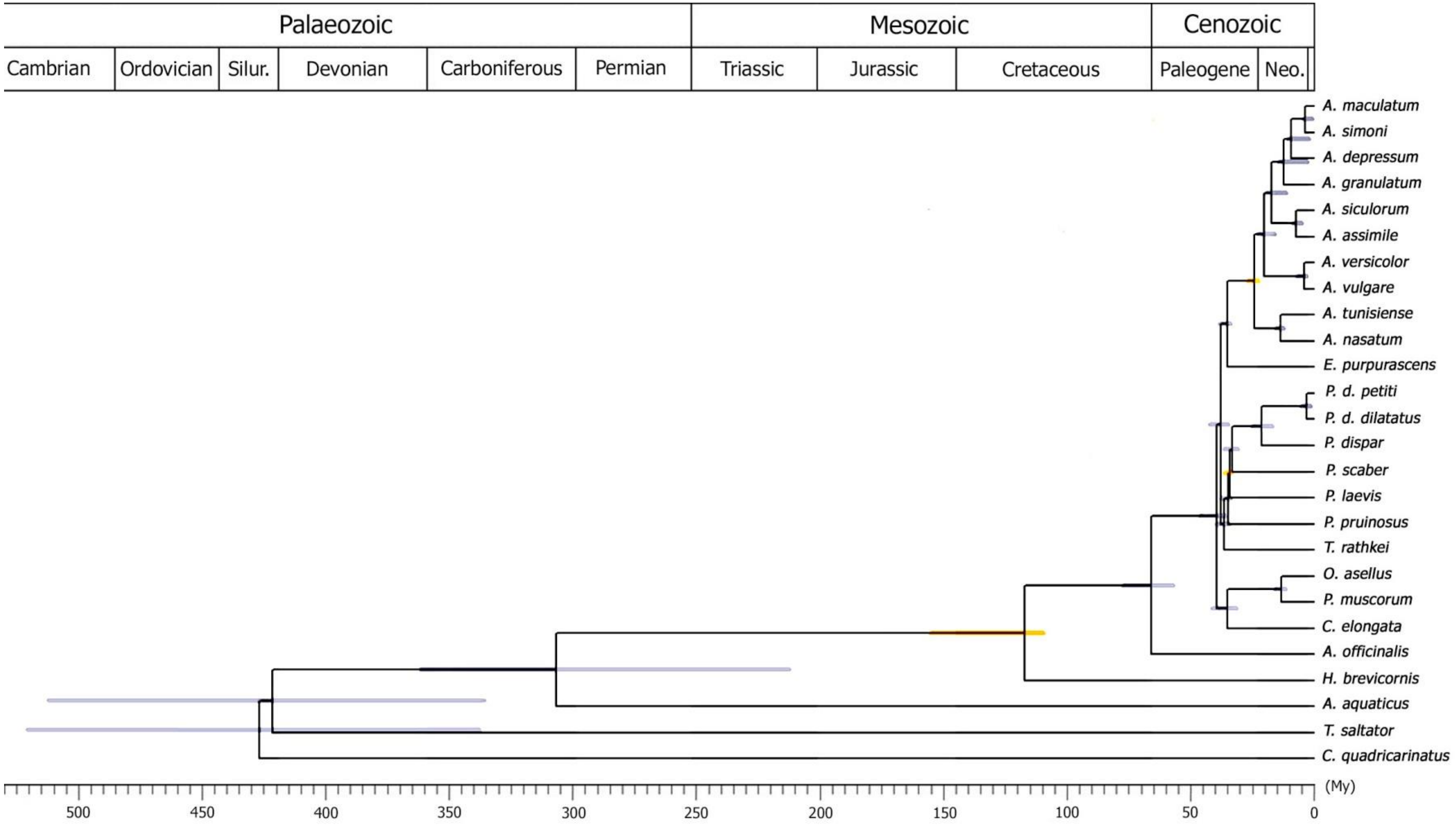

Supplementary Figure S2 : Chronogram of terrestrial isopod crustaceans, obtained using BEAST and the tree shown in Supplementary Figure S1 as a backbone. Branch length is scaled to time, with nodal ages corresponding to median posterior estimates. Grey bars represent the Highest Posterior Density (HPD) intervals at 95%. Orange bars denote the calibration points.

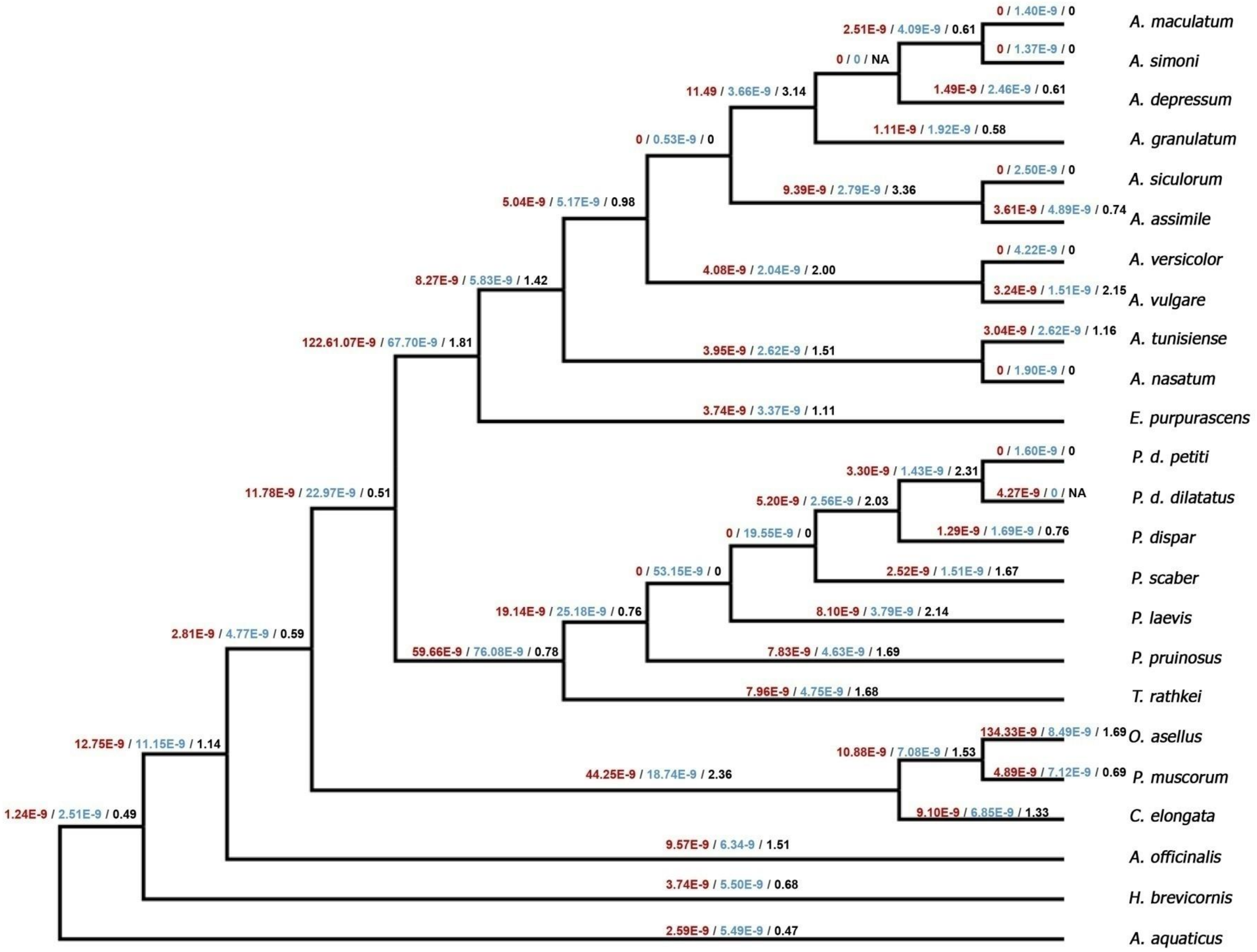

Figure S3. Cladogram indicating the silent substitution rate (dS) of each branch of isopods for mitochondrial sequences (red), nuclear sequences (blue) and the ratio between mitochondrial dS and nuclear dS (black). The per-site rate of amino acid replacements/ silent substitution rate ratio (dN/dS) of mitochondrial and nuclear dataset are estimated respectively at 0.2785 and 0.0674.

Supplementary Table S1. Information on transcriptomic data used.

Genus	Species	Origin (country)	Gender	Number of individuals used	Total number of raw reads	Accession number (source)
Armadillidium	*maculatum*	*Sainte Marguerite (France)*	♀	*1*	12,766,990	SRX2600479 (This study)
Armadillidium	*simoni*	*Villeneuve-loubet (France)*	♀	*1*	17,933,434	SRX2600482 (This study)
Armadillidium	*depressum*	*Saint Guilherm le Désert (France)*	♀	*1*	17,940,202	SRX2600477 (This study)
Armadillidium	*granulatum*	*Crete (Greece)*	♀	*1*	13,084,538	SRX2600478 (This study)
Armadillidium	*assimile*	*Saint-Maixent (France)*	♂	*1*	16,563,498	SRX2600476 (This study)
Armadillidium	*siculorum*	*Valle dei Templi (Sicily, Italy)*	♂	*1*	13,304,442	SRX26004781 (This study)
Armadillidium	*versicolor*	*Saint-Veit (Carynthia, Austria)*	♀	*1*	17,200,162	SRX2600484 (This study)
Armadillidium	*vulgare*	*?*	?	?	33,692,087	SRX564995 (Romiguier *et al.*, 2014)
Armadillidium	*nasatum*	*?*	?	?	15,552,480	SRX564993 and SRX564994 (Romiguier *et al.*, 2014)
Armadillidium	*tunisiense*	*Khniss Monastir (Tunisia)*	♀	*1*	24,948,176	SRX2600483 (This study)
Eluma	*purpurascens (caelatum)*	*Chizé (France)*	♀	*1*	14,402,732	SRX2600486 (This study)
Porcellionides	*pruinosus*	*Guadalquivir (Spain)*	♂	*1*	15,362,180	SRX2600492 (This study)
Porcellio	*laevis*	*Fréjus (France)*	♀	*1*	13,244,040	SRX2600491 (This study)
Porcellio	*scaber*	*Bois de la Borne (France)*	♂	*1*	16,303,962	SRX2600493 (This study)
Porcellio	*dispar*	*Santa Maria del Sol (Brasil)*	♀	*1*	15,455,100	SRX2600490 (This study)
Trachelipus	*rathkei*	*New York State (USA)*	1 ♀ and 1 ♂	*2*	237,910,854	SRR5198727 and SRR5198726 (This study)
Chaetophiloscia	*elongata*	*Turée (France)*	♂	*2*	16,269,144	SRX2600485 (This study)
Philoscia	*muscorum*	*Poitiers (France)*	♀	*1*	16,763,368	SRX2600489 (This study)
Oniscus	*asellus*	*Angers (France)*	♀	*1*	16,889,482	SRX2600488 (This study)
Armadillo	*officinalis*	*Crete (Greece)*	♀	*1*	15,903,154	SRX2600480 (This study)
Helleria	*brevicornis*	*Fréjus (France)*	♀	*1*	17,082,882	SRX2600486 (This study)
Asellus	*aquaticus*	*Planina cave (Slovenia)*	?	?	637,023	SRX1097282, SRX1097283, SRX1097286 and SRX1097290 (Speiser et al. 2014)
Talitrus	*saltator*	*Ynyslas beach (Wales, United Kingdom)*	♀ and ♂	*6*	49,459,410	SRX1896418 and SRX1896419 (O'Grady et al. 2016)
Cherax	*quadricarinatus*	*Darwin (Australia)*	*?*	*1*	32,344,260	ERX363978 (Tan et al. 2016)

Supplementary Table S2. Detailed information on sex reversal experiment success.

Species	number of injected females	number of injected individuals developing as females		number of dead individuals		number of putative neomales		number of attempted crosses	number of progenies
		n	%	n	%	n	%		
Armadillidium maculatum	12	2	16.67	4	33.33	6	50.00	18	8
Armadillidium depressum	7	1	14.29	2	28.57	4	57.14	12	7
Armadillidium granulatum	10	2	20.00	5	50.00	3	30.00	9	3
Armadillidium assimile	8	0	0.00	2	25.00	6	75.00	12	6
Armadillidium siculorum	4	2	50.00	0	0.00	2	50.00	4	1
Armadillidium versicolor	8	3	37.50	1	12.50	4	50.00	16	2
Armadillidium vulgare	10	2	20.00	4	40.00	4	40.00	8	1
Armadillidium nasatum	10	2	20.00	4	40.00	4	40.00	12	8
Armadillidium tunisiense	6	4	66.67	1	16.67	1	16.67	2	0
Porcellionides pruinosus	7	1	14.29	4	57.14	2	28.57	4	0
Porcellio scaber	9	3	33.33	4	44.44	2	22.22	4	2
Porcellio dispar	11	2	18.18	6	54.55	3	27.27	6	2
Chaetophiloscia elongata	7	0	0.00	7	100.00	0	0.00	0	0
Philoscia muscorum	8	0	0.00	8	100.00	0	0.00	0	0
Armadillo officinalis	18	1	5.56	8	44.44	9	50.00	18	3
**TOTAL :**	135	25		60		50		125	43

Supplementary Table S3. Statistical analyses of sex ratio from the broods obtained in crosses between females and neomales. Red cells indicate females infected by *Wolbachia* (excluded results). Orange cells indicate that the progeny sex-ratio is not significantly different from 50/50 (excluded results). Green cells indicate that the progeny sex-ratio is significantly different from 50/50 (included results).

Species		Male identification number	Progenies					Wolbachia ?	χ^2 Tests								Reference
			number of males		number of females		Total		Sex-ratio ≠ 50/50		Sex-ratio ≠ 66/33		Sex-ratio ≠ 75/25		Sex-ratio ≠ 100/0		
			n	%	n	%			X-squared	p-values	X-squared	p-values	X-squared	p-values	X-squared	p-values	
Armadillidium	*maculatum*	1	12	20.00	48	80.00	60	-	21.6	**3.36E-06**	4.8	**0.02846**	0.8	0.3711	∞	0	This study
Armadillidium	*maculatum*	2	10	55.56	8	44.44	18	-	0.2222	0.6374							This study
Armadillidium	*maculatum*	3	16	40.00	24	60.00	40	-	1.6	0.2059							This study
Armadillidium	*maculatum*	4	8	28.57	20	71.43	28	-	5.1429	**0.02334**	0.2857	0.593	0.1905	0.6625	∞	0	This study
Armadillidium	*maculatum*	4	9	21.43	33	78.57	42	-	13.7143	**0.0002128**	2.6786	0.1017	0.2857	0.593	∞	0	This study
Armadillidium	*maculatum*	4	7	20.00	28	80.00	35	-	12.6	**3.86E-04**	2.8	0.09426	0.4667	0.4945	∞	0	This study
Armadillidium	*maculatum*	4	5	31.25	11	68.75	16	-	2.25	0.1336							This study
Armadillidium	*maculatum*	4	4	22.22	14	77.78	18	-	5.5556	**0.01842**	1	0.3173	0.0741	0.7855	∞	0	This study
Armadillidium	*depressum*	1	11	20.75	42	79.25	53	-	18.1321	**2.06E-05**	3.7736	0.05207	0.5094	0.4754	∞	0	This study
Armadillidium	*depressum*	2	7	17.95	32	82.05	39	-	16.0256	**6.25E-05**	4.1538	**0.04154**	1.0342	0.3092	∞	0	This study
Armadillidium	*depressum*	2	3	21.43	11	78.57	14	+									This study
Armadillidium	*depressum*	3	13	22.03	46	77.97	59	+									This study
Armadillidium	*depressum*	3	7	16.67	35	83.33	42	-	18.6667	**1.56E-05**	5.25	**0.02195**	1.5556	0.2123	∞	0	This study
Armadillidium	*depressum*	4	7	31.82	15	68.18	22	-	2.9091	8.81E-02							This study
Armadillidium	*depressum*	4	7	41.18	10	58.82	17	+									This study
Armadillidium	*granulatum*	1	12	27.91	31	72.09	43	-	8.3953	**0.003762**	0.5698	0.4504	0.1938	0.6598	∞	0	This study
Armadillidium	*granulatum*	1	9	19.57	37	80.43	46	-	17.0435	**3.65E-05**	3.9239	**0.0476**	0.7246	0.3946	∞	0	This study
Armadillidium	*granulatum*	1	8	24.24	25	75.76	33	-	8.7576	**0.003083**	1.2273	0.2679	0.0101	0.9199	∞	0	This study

Armadillidium	*assimile*	1	25	100.00	0	0.00	25	-	25	**5.73E-07**	50	**1.54E-12**	75	**< 2.2e-16**	∞	0	This study
Armadillidium	*assimile*	1	6	100.00	0	0.00	6	-	6	**0.01431**	12	**0.000532**	18	**2.21E-05**	∞	0	This study
Armadillidium	*assimile*	2	20	100.00	0	0.00	20	-	20	**7.74E-06**	40	**2.54E-10**	60	**9.49E-15**	∞	0	This study
Armadillidium	*assimile*	3	60	100.00	0	0.00	60	-	60	**9.49E-15**	120	**< 2.2e-16**	180	**< 2.2e-16**	∞	0	This study
Armadillidium	*assimile*	3	14	100.00	0	0.00	14	-	14	**0.0001828**	28	**1.21E-07**	42	**9.13E-11**	∞	0	This study
Armadillidium	*assimile*	4	39	100.00	0	0.00	39	-	39	**4.24E-10**	78	**< 2.2e-16**	117	**< 2.2e-16**	∞	0	This study
Armadillidium	*siculorum*	1	12	40.00	18	60.00	30	-	1.2	0.2733							This study
Armadillidium	*versicolor*	1	7	58.33	5	41.67	12	-	0.3333	0.5637							This study
Armadillidium	*versicolor*	2	16	57.14	12	42.86	28	-	0.5714	0.4497							This study
Armadillidium	*vulgare*	1	3	25.00	9	75.00	12	-	3	0.08326							This study
Armadillidium	*vulgare*	1	64	28.57	160	71.43	224	NA	41.1429	**1.42E-10**	2.2857	0.1306	1.5238	0.217	∞	0	Juchault and Legrand, 1972
Armadillidium	*vulgare*	2	16	22.54	55	77.46	71	NA	21.4225	**3.68E-06**	3.7254	0.05359	0.23	0.6315	∞	0	Juchault and Legrand, 1972
Armadillidium	*vulgare*	3	39	23.35	128	76.65	167	NA	47.4311	**5.70E-12**	7.485	**0.006221**	0.2415	0.6231	∞	0	Juchault and Legrand, 1972
Armadillidium	*vulgare*	4	57	27.01	154	72.99	211	NA	44.5924	**2.43E-11**	3.7915	0.05151	0.4566	0.4992	∞	0	Juchault and Legrand, 1972
Armadillidium	*nasatum*	1	0	0.00	12	100.00	12	-	12	**0.000532**	6	**0.01431**	4	**0.0455**	NA	NA	This study
Armadillidium	*nasatum*	1	0	0.00	11	100.00	11	-	11	**0.0009111**	5.5	**0.01902**	3.6667	0.05551	NA	NA	This study
Armadillidium	*nasatum*	2	0	0.00	29	100.00	29	-	29	**7.24E-08**	14.5	**0.0001402**	9.6667	**0.001876**	NA	NA	This study
Armadillidium	*nasatum*	2	0	0.00	8	100.00	8	-	8	**0.004678**	4	**0.0455**	2.6667	0.1025	NA	NA	This study
Armadillidium	*nasatum*	3	7	58.33	5	41.67	12	-	0.3333	0.5637							This study
Armadillidium	*nasatum*	3	14	63.64	8	36.36	22	-	1.6364	0.2008							This study
Armadillidium	*nasatum*	4	0	0.00	29	100.00	29	-	29	**7.24E-08**	14.5	**1.40E-04**	9.6667	**0.001876**	NA	NA	This study
Armadillidium	*nasatum*	4	0	0.00	20	100.00	20	-	20	**7.74E-06**	10	**0.001565**	6.6667	**0.009823**	NA	NA	This study
Eluma	*purpurascens (caelatum)*	1	7	21.88	25	78.13	32	-	10.125	**0.001463**	1.8906	0.1691	0.1667	0.6831	∞	0	Juchault and Rigaud 1995
Eluma	*purpurascens (caelatum)*	2	2	16.67	10	83.33	12	-	5.3333	**0.02092**	1.5	0.2207	0.4444	0.505	∞	0	Juchault and Rigaud 1995
Eluma	*purpurascens (caelatum)*	3	7	28.00	18	72.00	25	-	4.84	**0.02781**	0.32	0.5716	0.12	0.729	∞	0	Juchault and Rigaud 1995

Eluma	*purpurascens (caelatum)*	4	11	22.92	37	77.08	48	-	14.0833	**0.0001749**	2.3438	0.1258	0.1111	0.7389	∞	0	Juchault and Rigaud 1995
Porcellio	*scaber*	1	9	30.00	21	70.00	30	-	4.8	**0.02846**	0.15	0.6985	0.4	0.5271	∞	0	This study
Porcellio	*scaber*	2	23	44.23	29	55.77	52	-	0.6923	0.4054							This study
Porcellio	*dispar*	1	20	64.52	11	35.48	31	+									This study
Porcellio	*dispar*	1	7	38.89	11	61.11	18	+									This study
Porcellio	*dilatatus petiti*	NA	185 (24)	23.30	609 (24)	76.70	794	NA	226.4181	**< 2.2e-16**	35.9704	**2.00E-09**	1.2242	0.2685	∞	0	Legrand *et al.*, 1974
Porcellio	*dilatatus dilatatus*	NA	0 (15)	0.00	207 (15)	100.00	207 (15)	NA	207	**< 2.2e-16**	103.5	**< 2.2e-16**	69	**< 2.2e-16**	NA	NA	Juchault and Legrand, 1964
Oniscus	*asellus*	1	5	21.74	18	78.26	23	-	7.3478	**0.006714**	1.3913	0.2382	0.1304	0.718	∞	0	Juchault and Rigaud 1995
Oniscus	*asellus*	2	3	16.67	15	83.33	18	-	8	**0.004678**	2.25	0.1336	0.6667	0.4142	∞	0	Juchault and Rigaud 1995
Oniscus	*asellus*	3	6	23.08	20	76.92	26	-	7.5385	**0.00604**	1.2308	0.2673	0.0513	0.8208	∞	0	Juchault and Rigaud 1995
Oniscus	*asellus*	4	5	18.52	22	81.48	27	-	10.7037	**0.001069**	2.6667	0.1025	0.6049	0.4367	∞	0	Juchault and Rigaud 1995
Oniscus	*asellus*	5	2	13.33	13	86.67	15	-	8.0667	**0.004509**	2.7	0.1003	1.0889	0.2967	∞	0	Juchault and Rigaud 1995
Oniscus	*asellus*	6	5	20.83	19	79.17	24	-	8.1667	**0.004267**	1.6875	0.1939	0.2222	0.6374	∞	0	Juchault and Rigaud 1995
Oniscus	*asellus*	7	7	25.00	21	75.00	28	-	9.8462	**0.001702**	2.3269	0.1272	0.4615	0.4969	∞	0	Juchault and Rigaud 1995
Armadillo	*officinalis*	1	0	0.00	17	100.00	17	-	17	**3.74E-05**	8.5	**0.003551**	5.6667	**0.01729**	NA	NA	This study
Armadillo	*officinalis*	2	0	0.00	18	100.00	18	-	18	**2.21E-05**	9	**0.0027**	6	**0.01431**	NA	NA	This study
Armadillo	*officinalis*	3	0	0.00	12	100.00	12	-	12	**0.000532**	6	**0.01431**	4	**0.0455**	NA	NA	This study
Helleria	*brevicornis*	NA	0 (2)	0.00	32 (2)	100.00	32 (2)	NA	32	**1.54E-08**	16	**6.33E-05**	10.6667	**0.001091**	NA	NA	Juchault and Legrand, 1964

Supplementary Table S4. Summary of the statistics used to determine heterogametic systems.

Species		Number of males (number of progenies)	Number of females (number of progenies)	Sex ratio (% of females / % of males)	χ^2 tests								Interpretation	Reference
					Sex-ratio $\neq$ 50/50		Sex-ratio $\neq$ 66/33		Sex-ratio $\neq$ 75/25		Sex-ratio $\neq$ 100/0			
					X-squared	p-values	X-squared	p-values	X-squared	p-values	X-squared	p-values		
Armadillidium	*maculatum*	40 (5)	143 (5)	78.1 / 21.9	57.9727	2.658E-14	10.8443	0.000991	0.9636	0.3263	∞	0	ZW/ZZ with viable WW genotype	This study
Armadillidium	*depressum*	25 (3)	109 (3)	81.3 / 18.7	52.6567	3.973E-13	12.9888	0.0003134	2.8756	0.08993	∞	0	ZW/ZZ with viable WW genotype	This study
Armadillidium	*granulatum*	29 (3)	93 (3)	76.2 / 23.8	33.5738	6.861E-09	5.0205	0.02505	0.0984	0.7538	∞	0	ZW/ZZ with viable WW genotype	This study
Armadillidium	*assimile*	164 (6)	0 (6)	0 / 100	164	< 2.2e-16	328	< 2.2e-16	492	< 2.2e-16	∞	0	?	This study
Armadillidium	*vulgare*	176 (4)	497 (4)	72.6 / 27.4	153.107	< 2.2e-16	15.6204	7.74E-05	0.476	0.4902	∞	0	ZW/ZZ with viable WW genotype	Juchault and Legrand, 1972
Armadillidium	*nasatum*	0 (6)	109 (6)	100 / 0	109	< 2.2e-16	54.5	1.554E-13	36.3333	1.663E-09	NA	NA	XY/XX	This study
Eluma	*purpurascens (caelatum)*	27 (4)	90 (4)	76.9 / 23.1	33.9231	5.73E-09	5.5385	0.0186	0.2308	0.631	∞	0	ZW/ZZ with viable WW genotype	Juchault and Rigaud, 1995
Porcellio	*scaber*	9 (1)	21 (1)	70 / 30	4.8	0.02846	0.15	0.6985	0.4	0.5271	∞	0	ZW/ZZ	This study
Porcellio	*dilatatus petiti*	185 (24)	609 (24)	76.7 / 23.3	226.4181	< 2.2e-16	35.9704	2.00E-09	1.2242	0.2685	∞	0	ZW/ZZ with viable WW genotype	Legrand *et al*, 1974
Porcellio	*dilatatus dilatatus*	0 (15)	207 (15)	100 / 0	207	< 2.2e-16	103.5	< 2.2e-16	69	< 2.2e-16	NA	NA	XY/XX	Juchault and Legrand, 1964
Armadillo	*officinalis*	0 (3)	47 (3)	100 / 0	47	7.10E-12	23.5	1.25E-06	15.6667	7.55E-05	NA	NA	XY/XX	This study
Oniscus	*asellus*	33 (7)	128 (7)	79.5 / 20.5	56.0559	7.04E-14	11.9379	0.00055	1.7412	0.187	∞	0	ZW/ZZ with viable WW genotype	Juchault and Rigaud, 1995
Helleria	*brevicornis*	0 (2)	32 (2)	100 / 0	32	1.54E-08	16	6.33E-05	10.6667	0.001091	NA	NA	XY/XX	Juchault and Legrand, 1964

Supplementary Table S5. Ancestral states reconstruction of the estimated number of transitions between sex determination systems. The numbers written for the maximum likelihood analysis correspond to the mean number of transitions estimated with 1,000 stochastic mappings (± standard deviation).

Types of discrete states used	Transition directions	Parsimony analysis	Maximum likelihood analysis	
			"ER" model	"SYM" model
A. assimile system treated as independent sex determination mechanism (1)	XY/XX to ZZ/ZW	1	1.66 (± 1.31)	3.54 (± 1.85)
	XY/XX to undetermined	0	1.04 (± 0.98)	0
	ZZ/ZW to XY/XX	2	4.79 (± 1.51)	6.86 (± 1.83)
	ZZ/ZW to undertermined	1	2.81 (± 1.28)	1.31 (± 0.54)
	undertermined to XY/XX	0	1.87 (± 1.23)	0
	undertermined to ZZ/ZW	0	1.36 (± 1.19)	0.13 (± 0.44)
	Total	4	13.53 (± 3.26)	11.84 (± 3.11)
A. assimile system treated as unknown mechanism (2)	XY/XX to ZZ/ZW	1	3.67 (± 1.94)	3.71 (± 1.91)
	ZZ/ZW to XY/XX	2	7.51 (± 1.83)	7.61 (± 1.84)
	Total	3	11.18 (± 3.08)	11.32 (± 3.07)

Supplementary Table S6. Metrics of the 19 isopod transcriptomes assembled in this study.

Species	Number of transcripts	Total size of assembly (nuc)	Mean transcript size (nuc)	Median transcript size (nuc)	N50 transcript length (nuc)
Armadillidium maculatum	49,506	31,897,914	644	380	928
Armadillidium simoni	53,420	37,351,839	699	381	1,112
Armadillidium depressum	57,394	42,641,755	743	381	1,283
Armadillidium granulatum	60,651	39,394,793	650	358	1,011
Armadillidium siculorum	60,934	37,141,038	610	346	884
Armadillidium assimile	56,481	37,002,359	655	378	995
Armadillidium versicolor	54,830	37,553,127	685	379	1,075
Armadillidium vulgare	112,787	50,040,006	444	234	755
Armadillidium tunisiense	77,764	52,869,378	680	343	1,179
Armadillidium nasatum	48,570	38,673,525	796	423	1,347
Eluma purpurascens (caelatum)	69,858	45,004,684	644	343	1,027
Porcellio dispar	65,731	41,771,358	635	352	961
Porcellio scaber	54,400	33,842,101	622	372	881
Porcellio laevis	48,428	29,369,984	606	376	821
Porcellionides pruinosus	67,260	46,231,902	687	369	1,114
Trachelipus rathkei	114,623	73,831,912	644	353	1,021
Oniscus asellus	61,150	39,337,685	643	367	953
Philoscia muscorum	64,025	44,470,959	695	379	1,115
Chaetophiloscia elongata	69,223	43,969,065	635	356	952
Armadillo officinalis	55,357	32,716,309	591	359	794
Helleria brevicornis	37,264	18,531,321	497	328	575
Asellus Aquaticus	21,684	11,582,160	534	464	506
Talitrus saltator	156,706	151,674,147	968	513	1,533
Cherax quadricarinatus	189,772	132,835,005	700	368	1,154

Supplementary Table S7. Detailed annotation of the 88 markers used in this study (5 first best BLASTX versus NCBI non-redundant database hit are shown).

Annotation						
	Best Blastp hits	Max score	Total score	Query cover	E value	Ident
**Ortho 1**	Methyl-CpG-binding domain protein 2 [Zootermopsis nevadensis]	372	372	97%	1.00E-126	69%
	methyl-CpG binding transcription regulator, putative [Ixodes scapularis]	349	349	95%	2.00E-117	64%
	PREDICTED: LOW QUALITY PROTEIN: methyl-CpG-binding domain protein 2-like [Limulus polyphemus]	344	344	95%	7.00E-116	65%
	PREDICTED: methyl-CpG-binding domain protein 2-like [Limulus polyphemus]	342	342	95%	8.00E-115	64%
	Methyl-CpG-binding domain protein 2 [Stegodyphus mimosarum]	333	333	94%	3.00E-111	62%
**Ortho 2**	innexin 1 [Homarus americanus]	562	562	99%	0	68%
	innexin 1 [Cancer borealis]	536	536	94%	0	67%
	innexin 1 [Schistocerca gregaria]	417	417	92%	1.00E-140	53%
	PREDICTED: innexin inx1 [Cimex lectularius]	417	417	92%	1.00E-140	52%
	RecName: Full=Innexin inx1; Short=Innexin-1; AltName: Full=G-Inx1 [Schistocerca americana]	416	416	92%	4.00E-140	53%
**Ortho 3**	PREDICTED: proteasome subunit alpha type-4 [Fopius arisanus]	424	424	95%	4.00E-147	78%
	PREDICTED: proteasome subunit alpha type-4 [Athalia rosae]	423	423	95%	1.00E-146	78%
	PREDICTED: proteasome subunit alpha type-4 [Cerapachys biroi]	422	422	95%	4.00E-146	78%
	PREDICTED: proteasome subunit alpha type-4 [Linepithema humile]	422	422	95%	4.00E-146	78%
	PREDICTED: proteasome subunit alpha type-4 [Dinoponera quadriceps]	421	421	95%	1.00E-145	78%
**Ortho 4**	PREDICTED: uncharacterized protein LOC106686239 [Halyomorpha halys]	99.4	99.4	78%	2.00E-20	29%
	conserved hypothetical protein [Riptortus pedestris]	94.4	94.4	72%	9.00E-19	29%
	PREDICTED: transmembrane protein 53 [Strongylocentrotus purpuratus]	93.2	93.2	86%	2.00E-18	26%
	Transmembrane protein 53 [Stegodyphus mimosarum]	90.9	90.9	85%	1.00E-17	28%
	hypothetical protein YQE_00956 [Dendroctonus ponderosae]	90.5	90.5	67%	2.00E-17	30%
**Ortho 5**	eukaryotic translation initiation factor 5A [Scylla paramamosain]	274	274	99%	5.00E-91	82%
	eukaryotic translation initiation factor 5 alpha [Procambarus clarkii]	270	270	99%	1.00E-89	80%
	eukaryotic translation initiation factor 5A [Penaeus monodon]	268	268	99%	1.00E-88	80%
	eukaryotic translation initiation factor 5A [Litopenaeus vannamei]	267	267	99%	2.00E-88	80%
	PREDICTED: eukaryotic translation initiation factor 5A [Stomoxys calcitrans]	242	242	98%	1.00E-78	71%
**Ortho 6**	AAEL011293-PA [Aedes aegypti]	144	144	44%	5.00E-38	48%
	PREDICTED: vitamin K epoxide reductase complex subunit 1-like protein 1 [Limulus polyphemus]	143	143	46%	1.00E-37	47%
	vitamin K epoxide reductase complex subunit 1 [Aedes aegypti]	143	143	44%	2.00E-37	48%
	vitamin K epoxide reductase complex subunit 1-like protein 1 [Danio rerio]	139	139	47%	7.00E-36	46%
	vitamin K epoxide reductase complex subunit 1-like protein 1 [Danio rerio]	139	139	47%	7.00E-36	46%
**Ortho 7**	PREDICTED: dolichol-phosphate mannosyltransferase subunit 1-like [Crassostrea gigas]	400	400	90%	7.00E-138	80%
	PREDICTED: probable dolichol-phosphate mannosyltransferase [Amyelois transitella]	399	399	91%	2.00E-137	78%
	PREDICTED: dolichol-phosphate mannosyltransferase subunit 1 [Lingula anatina]	397	397	91%	2.00E-136	77%
	PREDICTED: dolichyl-phosphate mannosyltransferase isoform X1 [Bombyx mori]	395	395	93%	9.00E-136	78%
	putative dolichol-phosphate mannosyltransferase [Danaus plexippus]	394	394	91%	2.00E-135	79%

Ortho 8	hypothetical protein DAPPUDRAFT_220118 [Daphnia pulex]	114	114	97%	4.00E-27	32%
	coiled-coil protein [Artemia franciscana]	95.1	95.1	97%	3.00E-20	28%
	PREDICTED: uncharacterized protein LOC105697737 isoform X1 [Orussus abietinus]	94.4	94.4	96%	9.00E-20	29%
	PREDICTED: uncharacterized protein LOC105697737 isoform X2 [Orussus abietinus]	94	94	96%	1.00E-19	29%
	Coiled-coil-helix-coiled-coil-helix domain-containing protein 3, mitochondrial precursor [Lepeophtheirus salmonis]	89.4	89.4	96%	5.00E-18	27%
Ortho 9	TMEM9 family protein [Coptotermes formosanus]	219	219	91%	4.00E-68	63%
	hypothetical protein L798_04988 [Zootermopsis nevadensis]	217	217	91%	2.00E-67	63%
	PREDICTED: uncharacterized protein CG1161 [Papilio polytes]	217	217	94%	2.00E-67	58%
	PREDICTED: uncharacterized protein CG1161 [Papilio machaon]	215	215	92%	1.00E-66	61%
	PREDICTED: uncharacterized protein CG1161 [Papilio xuthus]	213	213	81%	9.00E-66	67%
Ortho 10	PREDICTED: 28S ribosomal protein S21, mitochondrial [Halyomorpha halys]	115	115	78%	1.00E-30	57%
	AGAP009152-PA [Anopheles gambiae str. PEST]	114	114	75%	4.00E-30	57%
	mitochondrial 28S ribosomal protein S21 [Culex quinquefasciatus]	114	114	75%	8.00E-30	56%
	AAEL001909-PA [Aedes aegypti]	113	113	75%	1.00E-29	56%
	PREDICTED: 28S ribosomal protein S21, mitochondrial [Athalia rosae]	113	113	77%	1.00E-29	58%
Ortho 11	ribosomal protein L8 [Litopenaeus vannamei]	468	468	98%	2.00E-164	86%
	60S ribosomal protein L8 [Lepeophtheirus salmonis]	443	443	96%	1.00E-154	84%
	60S ribosomal protein L8 [Coptotermes formosanus]	442	442	97%	3.00E-154	82%
	PREDICTED: 60S ribosomal protein L8 [Athalia rosae]	441	441	98%	8.00E-154	81%
	60S ribosomal protein L8 [Laodelphax striatella]	440	440	97%	4.00E-153	83%
Ortho 12	PREDICTED: cold shock domain-containing protein CG9705-like [Plutella xylostella]	168	168	89%	2.00E-50	69%
	PREDICTED: cold shock domain-containing protein CG9705 [Bombyx mori]	166	166	89%	1.00E-49	70%
	PREDICTED: cold shock domain-containing protein CG9705 [Amyelois transitella]	166	166	89%	2.00E-49	70%
	calcium-regulated heat stable protein 1 [Acyrthosiphon pisum]	164	164	84%	8.00E-49	68%
	cold shock domain-containing protein CG9705 [Papilio xuthus]	161	161	89%	1.00E-47	68%
Ortho 13	conserved hypothetical protein [Pediculus humanus corporis]	223	223	82%	5.00E-67	45%
	Integral membrane protein 2B [Zootermopsis nevadensis]	222	222	95%	3.00E-66	41%
	PREDICTED: integral membrane protein 2B [Megachile rotundata]	204	204	95%	2.00E-59	38%
	PREDICTED: integral membrane protein 2C-like isoform X1 [Apis dorsata]	202	202	95%	1.00E-58	39%
	PREDICTED: integral membrane protein 2A [Tribolium castaneum]	201	201	95%	3.00E-58	42%
Ortho 14	PREDICTED: activator of 90 kDa heat shock protein ATPase homolog 1-like [Limulus polyphemus]	369	369	98%	1.00E-122	50%
	PREDICTED: activator of 90 kDa heat shock protein ATPase homolog 1 [Fopius arisanus]	360	360	98%	2.00E-119	53%
	PREDICTED: activator of 90 kDa heat shock protein ATPase homolog 1 [Halyomorpha halys]	358	358	98%	3.00E-118	51%
	PREDICTED: activator of 90 kDa heat shock protein ATPase homolog 1 isoform X1 [Acromyrmex echinatior]	356	356	98%	1.00E-117	52%
	hypothetical protein L798_08367 [Zootermopsis nevadensis]	356	356	98%	2.00E-117	51%
Ortho 15	adenine nucleotide translocase 2 [Litopenaeus vannamei]	559	559	94%	0	89%
	adenine nucleotide translocase [Litopenaeus vannamei]	555	555	95%	0	88%
	ATP/ADP translocase [Marsupenaeus japonicus]	548	548	94%	0	88%
	adenine nucleotide translocase [Penaeus monodon]	548	548	94%	0	88%
	ATP/ADP translocase [Pacifastacus leniusculus]	546	546	95%	0	88%
Ortho 16	V-ATPase A [Cherax destructor]	1112	1112	98%	0	84%
	V-H-ATPase subunit A [Cherax cainii]	1107	1107	98%	0	84%
	v-type proton atpase catalytic subunit a [Lasius niger]	1043	1043	96%	0	80%
	PREDICTED: V-type proton ATPase catalytic subunit A [Megachile rotundata]	1041	1041	96%	0	81%

	PREDICTED: V-type proton ATPase catalytic subunit A [Trichogramma pretiosum]	1040	1040	96%	0	80%
**Ortho 17**	ribosomal protein S9 [Procambarus clarkii]	337	337	91%	1.00E-114	92%
	hypothetical protein HELRODRAFT_155894 [Helobdella robusta]	322	322	94%	1.00E-108	85%
	ribosomal protein S9 [Riptortus pedestris]	322	322	96%	1.00E-108	81%
	PREDICTED: 40S ribosomal protein S9 [Halyomorpha halys]	320	320	96%	3.00E-108	81%
	S9e ribosomal protein [Meladema coriacea]	319	319	96%	9.00E-108	82%
**Ortho 18**	PREDICTED: 60S acidic ribosomal protein P2 [Thamnophis sirtalis]	78.2	78.2	95%	1.00E-15	45%
	60S acidic ribosomal protein P2 [Salmo salar]	77	77	95%	3.00E-15	50%
	PREDICTED: LOW QUALITY PROTEIN: ribosomal protein, large, P2 [Pantholops hodgsonii]	76.6	76.6	95%	4.00E-15	50%
	PREDICTED: 60S acidic ribosomal protein P2 isoform X2 [Anolis carolinensis]	76.3	76.3	95%	6.00E-15	49%
	hypothetical protein BN946_scf185008.g40 [Trametes cinnabarina]	75.5	75.5	95%	1.00E-14	45%
**Ortho 19**	hypothetical protein DAPPUDRAFT_300779 [Daphnia pulex]	624	624	90%	0	65%
	PREDICTED: nucleolar protein 58 isoform X1 [Apis florea]	619	619	87%	0	66%
	PREDICTED: nucleolar protein 58-like [Apis dorsata]	619	619	87%	0	66%
	PREDICTED: nucleolar protein 58 isoform X2 [Apis florea]	619	619	87%	0	66%
	PREDICTED: nucleolar protein 58-like [Apis mellifera]	618	618	87%	0	66%
**Ortho 20**	hypothetical protein DAPPUDRAFT_302032 [Daphnia pulex]	199	199	87%	1.00E-59	48%
	PREDICTED: ran-specific GTPase-activating protein [Microplitis demolitor]	197	197	65%	2.00E-58	56%
	Ran-specific GTPase-activating protein [Stegodyphus mimosarum]	196	196	81%	5.00E-58	54%
	PREDICTED: LOW QUALITY PROTEIN: ran-specific GTPase-activating protein-like [Bombus terrestris]	196	196	70%	7.00E-58	52%
	PREDICTED: ran-specific GTPase-activating protein-like [Bombus impatiens]	196	196	70%	9.00E-58	52%
**Ortho 21**	Aldo-keto reductase family 1 member B10 [Zootermopsis nevadensis]	474	474	96%	1.00E-164	67%
	PREDICTED: aldose reductase-like [Stomoxys calcitrans]	451	451	94%	2.00E-155	66%
	PREDICTED: aldose reductase-like isoform X2 [Vollenhovia emeryi]	449	449	97%	6.00E-155	64%
	PREDICTED: aldose reductase-like [Monomorium pharaonis]	449	449	97%	9.00E-155	65%
	prostaglandin F synthase [Penaeus monodon]	448	448	95%	2.00E-154	69%
**Ortho 22**	PREDICTED: LOW QUALITY PROTEIN: 60S ribosomal protein L18 [Solenopsis invicta]	304	304	98%	5.00E-101	75%
	PREDICTED: 60S ribosomal protein L18 [Wasmannia auropunctata]	304	304	98%	1.00E-100	76%
	PREDICTED: LOW QUALITY PROTEIN: 60S ribosomal protein L18 [Monomorium pharaonis]	302	302	98%	9.00E-100	75%
	PREDICTED: LOW QUALITY PROTEIN: 60S ribosomal protein L18 [Atta cephalotes]	300	300	98%	1.00E-99	75%
	hypothetical protein SINV_04174 [Solenopsis invicta]	296	296	96%	8.00E-99	75%
**Ortho 23**	muscle-specific protein 20 [Solenopsis invicta]	238	238	96%	3.00E-76	62%
	hypothetical protein SINV_13357 [Solenopsis invicta]	239	239	93%	3.00E-75	64%
	PREDICTED: muscle-specific protein 20 [Bombus terrestris]	234	234	96%	7.00E-75	61%
	PREDICTED: muscle-specific protein 20 [Bombus impatiens]	234	234	96%	8.00E-75	61%
	PREDICTED: muscle-specific protein 20 [Pogonomyrmex barbatus]	233	233	96%	2.00E-74	63%
**Ortho 24**	translationally controlled tumor protein [Marsupenaeus japonicus]	262	262	97%	4.00E-86	77%
	translationally controlled tumor protein [Fenneropenaeus chinensis]	261	261	97%	1.00E-85	78%
	translationally controlled tumor protein [Fenneropenaeus merguiensis]	261	261	97%	1.00E-85	77%
	translationally controlled tumor protein [Litopenaeus vannamei]	260	260	97%	3.00E-85	76%
	translationally controlled tumor protein [Marsupenaeus japonicus]	260	260	97%	4.00E-85	76%
**Ortho 25**	NADH dehydrogenase [ubiquinone] 1 beta subcomplex subunit 11, mitochondrial [Stegodyphus mimosarum]	112	112	73%	1.00E-27	49%
	mitochondrial NADH:ubiquinone oxidoreductase ESSS subunit, putative [Riptortus pedestris]	111	111	76%	2.00E-27	46%
	PREDICTED: NADH dehydrogenase [ubiquinone] 1 beta subcomplex subunit 11,	111	111	94%	3.00E-27	40%

	mitochondrial [Halyomorpha halys]					
	hypothetical protein CAPTEDRAFT_157472 [Capitella teleta]	111	111	94%	5.00E-27	37%
	PREDICTED: NADH dehydrogenase [ubiquinone] 1 beta subcomplex subunit 11, mitochondrial [Tribolium castaneum]	106	106	84%	1.00E-25	41%
**Ortho 26**	uncharacterized protein Dmoj_GI22699, isoform B [Drosophila mojavensis]	290	290	97%	1.00E-96	79%
	Cyclophilin-like [Drosophila melanogaster]	289	289	95%	1.00E-96	80%
	GI22699 [Drosophila mojavensis]	288	288	97%	2.00E-96	79%
	PREDICTED: peptidyl-prolyl cis-trans isomerase-like 1 [Ceratitis capitata]	288	288	95%	3.00E-96	81%
	PREDICTED: peptidyl-prolyl cis-trans isomerase-like 1 [Bactrocera oleae]	288	288	95%	3.00E-96	81%
**Ortho 27**	PREDICTED: p53 and DNA damage-regulated protein 1 [Latimeria chalumnae]	110	110	78%	4.00E-27	46%
	PREDICTED: p53 and DNA damage-regulated protein 1 [Clupea harengus]	101	101	78%	7.00E-24	43%
	p53 and DNA damage-regulated protein 1 [Anoplopoma fimbria]	100	100	76%	1.00E-23	41%
	PREDICTED: p53 and DNA damage-regulated protein 1 isoform X1 [Cynoglossus semilaevis]	100	100	78%	2.00E-23	41%
	PREDICTED: p53 and DNA damage-regulated protein 1 [Larimichthys crocea]	99.4	99.4	78%	6.00E-23	42%
**Ortho 28**	Regulator of G-protein signaling 3 [Stegodyphus mimosarum]	197	197	76%	2.00E-59	60%
	PREDICTED: regulator of G-protein signaling 2-like [Limulus polyphemus]	194	194	76%	9.00E-59	58%
	PREDICTED: regulator of G-protein signaling 12-like [Limulus polyphemus]	198	198	88%	7.00E-58	55%
	PREDICTED: regulator of G-protein signaling 3-like [Limulus polyphemus]	195	195	74%	1.00E-56	62%
	PREDICTED: regulator of G-protein signaling 2-like [Limulus polyphemus]	188	188	74%	3.00E-56	60%
**Ortho 29**	core histone H2A/H2B/H3/H4 [Dictyocaulus viviparus]	275	275	98%	4.00E-92	99%
	PREDICTED: LOW QUALITY PROTEIN: histone H3.3 [Felis catus]	275	275	97%	6.00E-92	100%
	Histone H3.3 [Habropoda laboriosa]	274	274	98%	1.00E-91	99%
	histone H3.3 [Homo sapiens]	273	273	97%	1.00E-91	100%
	Chain A, The Human Nucleosome Structure Containing The Histone Variant H3.3	273	273	97%	2.00E-91	100%
**Ortho 30**	ribonucleotide reductase, beta subunit, putative [Ixodes scapularis]	555	555	96%	0	73%
	PREDICTED: ribonucleoside-diphosphate reductase subunit M2 B-like [Limulus polyphemus]	537	537	98%	0	72%
	Ribonucleoside-diphosphate reductase subunit M2 [Stegodyphus mimosarum]	535	535	100%	0	71%
	ribonucleotide reductase small subunit R2ii [Carassius carassius]	535	535	98%	0	72%
	PREDICTED: ribonucleoside-diphosphate reductase subunit M2 B isoform X2 [Halyomorpha halys]	533	533	100%	0	71%
**Ortho 31**	cathepsin B (Marsupenaeus japonicus)	433	433	96%	3.00E-148	60%
	cathepsin B (Fenneropenaeus chinensis)	432	432	95%	7.00E-148	60%
	cathepsin B (Litopenaeus vannamei)	432	432	91%	1.00E-147	63%
	cathepsin B (Palaemon carinicauda)	430	430	99%	8.00E-147	59%
	cathepsin B (Penaeus monodon)	429	429	95%	9.00E-147	60%
**Ortho 32**	PREDICTED: MFS-type transporter SLC18B1-like (Trichogramma pretiosum)	488	488	97%	1.00E-164	54%
	uncharacterized protein LOC100123296 precursor (Nasonia vitripennis)	479	479	89%	2.00E-161	56%
	Chromaffin granule amine transporter, putative (Pediculus humanus corporis)	474	474	85%	3.00E-159	56%
	PREDICTED: MFS-type transporter SLC18B1-like (Solenopsis invicta)	472	472	88%	2.00E-158	55%
	PREDICTED: MFS-type transporter SLC18B1-like (Monomorium pharaonis)	471	471	88%	3.00E-158	55%
**Ortho 33**	PREDICTED: cytochrome b-c1 complex subunit 2, mitochondrial (Megachile rotundata)	304	304	91%	2.00E-94	42%
	Cytochrome b-c1 complex subunit 2, mitochondrial (Habropoda laboriosa)	299	299	91%	1.00E-92	41%
	cytochrome b-c1 complex subunit mitochondrial (Lasius niger)	293	293	99%	5.00E-90	40%
	PREDICTED: cytochrome b-c1 complex subunit 2, mitochondrial (Acromyrmex echinatior)	289	289	99%	1.00E-88	39%
	Ubiquinol-cytochrome C reductase complex (Xenopus laevis)	289	289	92%	2.00E-88	40%
**Ortho**	PREDICTED: probable dynactin subunit 2 (Linepithema humile)	307	307	98%	9.00E-97	41%

34	putative dynactin subunit 2 (Zootermopsis nevadensis)	302	302	99%	7.00E-95	42%
	unknown (Dendroctonus ponderosae)	300	300	99%	3.00E-94	42%
	PREDICTED: probable dynactin subunit 2 (Camponotus floridanus)	301	301	98%	3.00E-94	40%
	PREDICTED: probable dynactin subunit 2 (Monomorium pharaonis)	297	297	98%	8.00E-93	40%
**Ortho 35**	PREDICTED: NADH-ubiquinone oxidoreductase 49 kDa subunit (Bactrocera dorsalis)	738	738	96%	0	74%
	PREDICTED: NADH-ubiquinone oxidoreductase 49 kDa subunit (Bactrocera oleae)	737	737	96%	0	74%
	PREDICTED: NADH-ubiquinone oxidoreductase 49 kDa subunit (Bactrocera cucurbitae)	736	736	96%	0	74%
	GH23924 (Drosophila grimshawi)	733	733	90%	0	79%
	PREDICTED: NADH-ubiquinone oxidoreductase 49 kDa subunit (Ceratitis capitata)	733	733	91%	0	78%
**Ortho 36**	PREDICTED: transmembrane protein 165 isoform X1 (Plutella xylostella)	282	282	75%	1.00E-89	56%
	PREDICTED: transmembrane protein 165 isoform X2 (Plutella xylostella)	281	281	80%	2.00E-89	54%
	PREDICTED: transmembrane protein 165 (Microplitis demolitor)	280	280	75%	8.00E-89	56%
	PREDICTED: transmembrane protein 165-like isoform X4 (Halyomorpha halys)	278	278	73%	1.00E-88	56%
	PREDICTED: transmembrane protein 165 isoform X3 (Megachile rotundata)	278	278	78%	1.00E-88	53%
**Ortho 37**	Xaa-Pro dipeptidase (Zootermopsis nevadensis)	303	303	70%	5.00E-87	34%
	PREDICTED: FAST kinase domain-containing protein 3-like (Limulus polyphemus)	253	253	97%	6.00E-70	26%
	AAEL008901-PA (Aedes aegypti)	243	243	92%	1.00E-66	27%
	PREDICTED: uncharacterized protein LOC105205340 (Solenopsis invicta)	236	236	91%	7.00E-64	28%
	conserved hypothetical protein (Culex quinquefasciatus)	233	233	92%	7.00E-63	28%
**Ortho 38**	PREDICTED: uncharacterized protein LOC106174438 isoform X2 (Lingula anatina)	275	275	94%	1.00E-82	56%
	PREDICTED: uncharacterized protein LOC106174438 isoform X5 (Lingula anatina)	273	273	82%	4.00E-82	61%
	PREDICTED: uncharacterized protein LOC106174438 isoform X4 (Lingula anatina)	273	273	82%	4.00E-82	61%
	PREDICTED: uncharacterized protein LOC106174438 isoform X1 (Lingula anatina)	274	274	82%	5.00E-82	61%
	PREDICTED: uncharacterized protein LOC106174438 isoform X3 (Lingula anatina)	273	273	82%	6.00E-82	61%
**Ortho 40**	ADP-ribosylation factor-like protein 8B-A (Zootermopsis nevadensis)	374	374	98%	1.00E-129	96%
	PREDICTED: ADP-ribosylation factor-like protein 8 (Tribolium castaneum)	372	372	98%	1.00E-128	95%
	PREDICTED: ADP-ribosylation factor-like protein 8B-A (Acromyrmex echinatior)	371	371	98%	2.00E-128	96%
	PREDICTED: ADP-ribosylation factor-like protein 8B-A (Trichogramma pretiosum)	370	370	98%	3.00E-128	95%
	PREDICTED: ADP-ribosylation factor-like protein 8B-A (Nasonia vitripennis)	370	370	98%	3.00E-128	95%
**Ortho 41**	40S ribosomal protein S6 (Zootermopsis nevadensis)	408	408	42%	6.00E-136	79%
	PREDICTED: LOW QUALITY PROTEIN: 40S ribosomal protein S6 (Linepithema humile)	405	405	42%	6.00E-135	78%
	ribosomal protein S6 (Riptortus pedestris)	404	404	42%	3.00E-134	78%
	40s ribosomal protein s6-like protein (Lasius niger)	403	403	42%	6.00E-134	77%
	PREDICTED: 40S ribosomal protein S6 (Camponotus floridanus)	403	403	42%	8.00E-134	77%
**Ortho 42**	Thioredoxin domain-containing protein 15 (Papilio xuthus)	117	117	50%	2.00E-27	46%
	PREDICTED: thioredoxin domain-containing protein 15 (Papilio xuthus)	117	117	50%	3.00E-27	46%
	Thioredoxin domain-containing protein 15 (Papilio machaon)	117	117	50%	4.00E-27	45%
	PREDICTED: thioredoxin domain-containing protein 15 (Papilio machaon)	117	117	50%	4.00E-27	45%
	thioredoxin domain-containing protein 15 precursor (Papilio xuthus)	116	116	50%	4.00E-27	46%
**Ortho 43**	PREDICTED: tribbles homolog 2 (Tribolium castaneum)	256	256	79%	8.00E-79	49%
	PREDICTED: tribbles homolog 2 (Cimex lectularius)	250	250	78%	1.00E-76	48%
	PREDICTED: tribbles homolog 2 (Atta cephalotes)	250	250	87%	4.00E-76	47%
	PREDICTED: tribbles homolog 2 (Orussus abietinus)	249	249	88%	5.00E-76	45%
	PREDICTED: tribbles homolog 2 (Plutella xylostella)	249	249	79%	6.00E-76	51%

**Ortho 44**	troponin C2 (Litopenaeus vannamei)	234	234	98%	2.00E-75	79%
	troponin C isoform 4' (Homarus americanus)	229	229	96%	2.00E-73	77%
	troponin C (Crangon crangon)	224	224	98%	2.00E-71	74%
	troponin C isoform 3 (Homarus americanus)	223	223	98%	2.00E-71	75%
	troponin C1 (Litopenaeus vannamei)	220	220	98%	4.00E-70	74%
**Ortho 45**	N(G),N(G)-dimethylarginine dimethylaminohydrolase 1 (Stegodyphus mimosarum)	327	327	97%	2.00E-108	56%
	PREDICTED: N(G),N(G)-dimethylarginine dimethylaminohydrolase 1-like (Limulus polyphemus)	324	324	97%	4.00E-107	58%
	PREDICTED: N(G),N(G)-dimethylarginine dimethylaminohydrolase 1-like (Limulus polyphemus)	318	318	97%	6.00E-105	58%
	PREDICTED: LOW QUALITY PROTEIN: N(G),N(G)-dimethylarginine dimethylaminohydrolase 1-like (Limulus polyphemus)	310	310	94%	8.00E-102	56%
	PREDICTED: N(G),N(G)-dimethylarginine dimethylaminohydrolase 1 (Trichogramma pretiosum)	299	299	97%	2.00E-97	56%
**Ortho 46**	arginine kinase (Litopenaeus vannamei)	687	687	96%	0	90%
	arginine kinase (Fenneropenaeus merguiensis)	686	686	96%	0	90%
	arginine kinase (Metapenaeus ensis)	686	686	96%	0	90%
	arginine kinase (Litopenaeus vannamei)	685	685	96%	0	90%
	RecName: Full=Arginine kinase; Short=AK; AltName: Allergen=Pen m 2 (Penaeus monodon)	685	685	96%	0	90%
**Ortho 47**	PREDICTED: phospholipase A1 member A (Nannospalax galili)	104	104	77%	5.00E-22	33%
	PREDICTED: phospholipase A1 member A isoform X2 (Camelus bactrianus)	100	100	77%	6.00E-21	32%
	PREDICTED: phospholipase A1 member A isoform X2 (Camelus dromedarius)	100	100	77%	7.00E-21	32%
	PREDICTED: phospholipase A1 member A isoform X2 (Camelus ferus)	100	100	77%	1.00E-20	32%
	PREDICTED: phospholipase A1 member A (Dipodomys ordii)	100	100	77%	2.00E-20	32%
**Ortho 48**	PREDICTED: stromal membrane-associated protein 1 (Amyelois transitella)	327	327	77%	1.00E-103	49%
	PREDICTED: stromal membrane-associated protein 1 (Solenopsis invicta)	331	331	75%	1.00E-103	51%
	PREDICTED: stromal membrane-associated protein 1 (Papilio polytes)	327	327	64%	4.00E-103	53%
	PREDICTED: stromal membrane-associated protein 1 (Camponotus floridanus)	328	328	75%	4.00E-103	51%
	PREDICTED: stromal membrane-associated protein 1 (Wasmannia auropunctata)	329	329	88%	1.00E-102	45%
**Ortho 49**	C11orf46 homolog (Caligus clemensi)	93.6	93.6	31%	1.00E-19	45%
	PREDICTED: ARL14 effector protein (Amyelois transitella)	92.8	92.8	27%	1.00E-19	49%
	Cdc2c (Operophtera brumata)	91.7	91.7	27%	3.00E-19	47%
	PREDICTED: ARL14 effector protein (Papilio polytes)	90.9	90.9	24%	6.00E-19	52%
	PREDICTED: ARL14 effector protein (Papilio machaon)	90.5	90.5	24%	6.00E-19	50%
**Ortho 50**	lethal 35Di (Culex quinquefasciatus)	160	160	81%	3.00E-46	53%
	lethal 35Di (Anopheles darlingi)	157	157	81%	6.00E-45	52%
	PREDICTED: NADH dehydrogenase (ubiquinone) 1 beta subcomplex subunit 6 (Amyelois transitella)	154	154	82%	1.00E-43	51%
	PREDICTED: NADH dehydrogenase (ubiquinone) 1 beta subcomplex subunit 6 (Fopius arisanus)	154	154	81%	2.00E-43	50%
	AAEL009066-PA (Aedes aegypti)	152	152	81%	5.00E-43	52%
**Ortho 51**	PREDICTED: stromal cell-derived factor 2 (Trichogramma pretiosum)	252	252	99%	1.00E-80	53%
	PREDICTED: stromal cell-derived factor 2 (Tribolium castaneum)	251	251	85%	4.00E-80	59%
	Stromal cell-derived factor 2 (Zootermopsis nevadensis)	252	252	84%	3.00E-79	63%
	hypothetical protein DAPPUDRAFT_227363 (Daphnia pulex)	247	247	92%	2.00E-78	56%
	PREDICTED: stromal cell-derived factor 2 (Harpegnathos saltator)	247	247	85%	2.00E-78	56%
**Ortho 52**	PREDICTED: actin-related protein 2/3 complex subunit 2 (Bactrocera dorsalis)	472	472	97%	1.00E-164	75%
	PREDICTED: actin-related protein 2/3 complex subunit 2 (Bactrocera cucurbitae)	471	471	97%	4.00E-164	75%
	putative actin-related protein 2/3 complex subunit 2 (Zootermopsis nevadensis)	470	470	96%	1.00E-163	75%
	PREDICTED: actin-related protein 2/3 complex subunit 2 (Ceratitis capitata)	470	470	97%	1.00E-163	75%

	PREDICTED: actin-related protein 2/3 complex subunit 2 (Bactrocera oleae)	469	469	97%	2.00E-163	75%
**Ortho 53**	hypothetical protein AMK59_1816 (Oryctes borbonicus)	296	296	98%	5.00E-97	58%
	Proteasome activator complex subunit 3 (Stegodyphus mimosarum)	296	296	96%	5.00E-97	58%
	PREDICTED: proteasome activator complex subunit 3 isoform X2 (Musca domestica)	295	295	96%	2.00E-96	58%
	LOW QUALITY PROTEIN: uncharacterized protein Dmoj_GI15909 (Drosophila mojavensis)	293	293	98%	6.00E-96	57%
	PREDICTED: proteasome activator complex subunit 3 isoform X1 (Musca domestica)	293	293	97%	1.00E-95	57%
**Ortho 54**	PREDICTED: uncharacterized protein C19orf60 homolog (Limulus polyphemus)	113	113	75%	5.00E-28	46%
	fed tick salivary protein 6 (Ixodes scapularis)	108	108	79%	3.00E-26	43%
	secreted salivary gland peptide, putative (Ixodes scapularis)	109	109	79%	4.00E-26	43%
	PREDICTED: uncharacterized protein C19orf60 homolog (Lingula anatina)	105	105	76%	8.00E-25	42%
	PREDICTED: uncharacterized protein C19orf60 homolog (Meleagris gallopavo)	105	105	75%	1.00E-24	41%
**Ortho 55**	PREDICTED: synaptosomal-associated protein 29 (Amyelois transitella)	172	172	95%	1.00E-47	40%
	Synaptosomal-associated protein 29 (Zootermopsis nevadensis)	168	168	84%	3.00E-46	42%
	hypothetical protein DAPPUDRAFT_194582 (Daphnia pulex)	165	165	80%	5.00E-45	43%
	PREDICTED: soluble NSF attachment protein 29 (Bombyx mori)	163	163	98%	2.00E-44	38%
	PREDICTED: synaptosomal-associated protein 29 (Papilio machaon)	162	162	98%	3.00E-44	38%
**Ortho 56**	mitochondrial cytochrome c oxidase subunit Va (Litopenaeus vannamei)	229	229	95%	2.00E-73	68%
	PREDICTED: cytochrome c oxidase subunit 5A, mitochondrial (Tribolium castaneum)	223	223	96%	5.00E-71	67%
	AAEL014944-PA (Aedes aegypti)	218	218	96%	2.00E-69	67%
	cytochrome c oxidase polypeptide (Anopheles darlingi)	216	216	96%	2.00E-68	66%
	Cytochrome c oxidase subunit 5A, mitochondrial (Zootermopsis nevadensis)	216	216	96%	3.00E-68	64%
**Ortho 57**	Pinx1 (Litopenaeus vannamei)	172	172	29%	2.00E-46	51%
	PREDICTED: PIN2/TERF1-interacting telomerase inhibitor 1 (Fundulus heteroclitus)	176	176	40%	2.00E-45	42%
	Pin2-interacting protein X1 (Esox lucius)	170	170	36%	4.00E-45	42%
	PREDICTED: PIN2/TERF1-interacting telomerase inhibitor 1 (Esox lucius)	172	172	36%	3.00E-44	42%
	PREDICTED: PIN2/TERF1-interacting telomerase inhibitor 1 (Clupea harengus)	169	169	40%	5.00E-43	42%
**Ortho 58**	Tubulin-specific chaperone cofactor E-like protein (Cerapachys biroi)	365	365	99%	2.00E-118	46%
	Tubulin-specific chaperone cofactor E-like protein (Habropoda laboriosa)	357	357	99%	3.00E-115	45%
	PREDICTED: tubulin-specific chaperone cofactor E-like protein isoform X2 (Cimex lectularius)	347	347	99%	1.00E-111	43%
	PREDICTED: tubulin-specific chaperone cofactor E-like protein isoform X1 (Cimex lectularius)	348	348	99%	2.00E-111	43%
	PREDICTED: tubulin-specific chaperone cofactor E-like protein (Plutella xylostella)	341	341	99%	5.00E-109	42%
**Ortho 59**	nucleotide excision repair protein (Marsupenaeus japonicus)	423	423	98%	9.00E-143	65%
	UV excision repair protein RAD23-like protein B (Zootermopsis nevadensis)	360	360	98%	4.00E-118	52%
	PREDICTED: UV excision repair protein RAD23 homolog B-like (Limulus polyphemus)	343	343	98%	3.00E-111	52%
	uv excision repair protein rad23, putative (Pediculus humanus corporis)	338	338	98%	8.00E-110	49%
	PREDICTED: UV excision repair protein RAD23 homolog B (Orussus abietinus)	337	337	98%	3.00E-109	49%
**Ortho 61**	PREDICTED: charged multivesicular body protein 2b (Bombyx mori)	254	254	94%	1.00E-81	60%
	vacuolar assembly/sorting protein DID4, putative (Ixodes scapularis)	254	254	93%	2.00E-81	60%
	PREDICTED: charged multivesicular body protein 2b (Amyelois transitella)	253	253	93%	3.00E-81	60%
	PREDICTED: charged multivesicular body protein 2b-B (Papilio xuthus)	251	251	93%	4.00E-80	58%
	PREDICTED: charged multivesicular body protein 2b-B (Plutella xylostella)	249	249	93%	1.00E-79	60%
**Ortho 62**	PREDICTED: DNA-directed RNA polymerases I and III subunit RPAC1-like (Lingula anatina)	317	317	85%	2.00E-102	52%
	hypothetical protein DAPPUDRAFT_213644 (Daphnia pulex)	310	310	84%	2.00E-99	52%
	DNA-directed RNA polymerases I and III subunit RPAC1 (Zootermopsis nevadensis)	307	307	84%	1.00E-98	51%

	PREDICTED: DNA-directed RNA polymerases I and III subunit RPAC1-like isoform X1 (Limulus polyphemus)	307	307	87%	2.00E-98	52%
	PREDICTED: DNA-directed RNA polymerases I and III subunit RPAC1 (Bombyx mori)	305	305	82%	8.00E-98	54%
**Ortho 63**	PREDICTED: methylmalonic aciduria and homocystinuria type D homolog, mitochondrial-like (Chrysochloris asiatica)	214	214	87%	3.00E-63	41%
	PREDICTED: methylmalonic aciduria and homocystinuria type D protein, mitochondrial (Erinaceus europaeus)	213	213	88%	4.00E-63	41%
	PREDICTED: methylmalonic aciduria and homocystinuria type D protein, mitochondrial (Loxodonta africana)	213	213	87%	7.00E-63	42%
	PREDICTED: methylmalonic aciduria and homocystinuria type D protein, mitochondrial (Condylura cristata)	212	212	87%	2.00E-62	41%
	PREDICTED: methylmalonic aciduria and homocystinuria type D homolog, mitochondrial-like (Bubalus bubalis)	211	211	87%	3.00E-62	42%
**Ortho 64**	E3 ubiquitin-protein ligase TM129 (Xenopus laevis)	303	303	93%	4.00E-96	42%
	E3 ubiquitin-protein ligase TM129 (Xenopus (Silurana) tropicalis)	301	301	93%	2.00E-95	42%
	PREDICTED: transmembrane protein 129 (Chrysemys picta bellii)	296	296	93%	2.00E-93	40%
	E3 ubiquitin-protein ligase TM129 isoform X1 (Amazona aestiva)	296	296	93%	2.00E-93	42%
	PREDICTED: E3 ubiquitin-protein ligase TM129 isoform X1 (Falco peregrinus)	295	295	93%	6.00E-93	41%
**Ortho 65**	unknown (Dendroctonus ponderosae)	377	377	98%	6.00E-124	54%
	PREDICTED: zinc finger CCCH domain-containing protein 15 homolog (Fopius arisanus)	372	372	94%	5.00E-122	55%
	PREDICTED: zinc finger CCCH domain-containing protein 15 homolog (Monomorium pharaonis)	364	364	94%	7.00E-119	56%
	PREDICTED: zinc finger CCCH domain-containing protein 15 homolog (Atta cephalotes)	362	362	88%	5.00E-118	55%
	PREDICTED: zinc finger CCCH domain-containing protein 15 homolog (Acromyrmex echinatior)	362	362	88%	7.00E-118	56%
**Ortho 66**	PREDICTED: coiled-coil domain-containing protein 86-like (Salmo salar)	104	104	76%	2.00E-24	50%
	unnamed protein product (Oncorhynchus mykiss)	106	106	76%	2.00E-24	50%
	Coiled-coil domain-containing protein 86 (Melipona quadrifasciata)	101	101	63%	2.00E-23	51%
	PREDICTED: coiled-coil domain-containing protein 86 (Esox lucius)	103	103	75%	5.00E-23	50%
	PREDICTED: coiled-coil domain-containing protein 86 (Wasmannia auropunctata)	100	100	72%	6.00E-23	46%
**Ortho 67**	hypothetical protein SINV_14247 (Solenopsis invicta)	157	157	88%	1.00E-45	81%
	TPA: alternative splicing factor ASF/SF2 (Amblyomma variegatum)	157	157	82%	4.00E-45	89%
	Splicing factor, arginine/serine-rich 1 (Harpegnathos saltator)	155	155	87%	6.00E-45	82%
	PREDICTED: serine/arginine-rich splicing factor 1B (Megachile rotundata)	156	156	87%	9.00E-45	82%
	PREDICTED: serine/arginine-rich splicing factor 1A (Orussus abietinus)	156	156	87%	1.00E-44	82%
**Ortho 68**	PREDICTED: bridging integrator 3-like (Athalia rosae)	88.2	88.2	91%	5.00E-17	26%
	PREDICTED: bridging integrator 3-like (Solenopsis invicta)	83.6	83.6	91%	1.00E-15	25%
	PREDICTED: bridging integrator 3-like (Monomorium pharaonis)	83.2	83.2	91%	2.00E-15	25%
	PREDICTED: bridging integrator 3-like isoform X1 (Cerapachys biroi)	83.2	83.2	91%	2.00E-15	25%
	PREDICTED: bridging integrator 3 homolog (Harpegnathos saltator)	83.2	83.2	91%	2.00E-15	26%
**Ortho 69**	hypothetical protein CAPTEDRAFT_152195 (Capitella teleta)	231	231	99%	1.00E-72	51%
	hypothetical protein LOTGIDRAFT_222883 (Lottia gigantea)	229	229	99%	9.00E-72	52%
	PREDICTED: Golgi SNAP receptor complex member 2-like (Limulus polyphemus)	216	216	99%	2.00E-66	49%
	PREDICTED: Golgi SNAP receptor complex member 2-like (Biomphalaria glabrata)	212	212	99%	4.00E-65	50%
	PREDICTED: Golgi SNAP receptor complex member 2-like isoform X1 (Saccoglossus kowalevskii)	211	211	99%	6.00E-65	53%
**Ortho 70**	NEDD8-conjugating enzyme Ubc12 (Zootermopsis nevadensis)	325	325	96%	2.00E-110	85%
	PREDICTED: NEDD8-conjugating enzyme Ubc12 (Fopius arisanus)	322	322	96%	5.00E-109	85%
	NEDD8-conjugating enzyme Ubc12, putative (Pediculus humanus corporis)	321	321	96%	1.00E-108	83%
	PREDICTED: NEDD8-conjugating enzyme Ubc12 (Monomorium pharaonis)	320	320	96%	3.00E-108	85%
	hypothetical protein CAPTEDRAFT_149080 (Capitella teleta)	320	320	96%	3.00E-108	83%
**Ortho**	OTU domain ubiquitin aldehyde binding protein (Scylla paramamosain)	392	392	84%	8.00E-134	73%

71	OTU domain, ubiquitin aldehyde binding protein, putative (Ixodes scapularis)	276	276	85%	5.00E-88	53%
	PREDICTED: ubiquitin thioesterase OTUB1-like isoform X1 (Limulus polyphemus)	276	276	84%	7.00E-88	53%
	PREDICTED: ubiquitin thioesterase otubain-like (Fopius arisanus)	273	273	88%	4.00E-87	50%
	PREDICTED: ubiquitin thioesterase otubain-like (Microplitis demolitor)	273	273	88%	5.00E-87	49%
Ortho 72	Ribosomal RNA small subunit methyltransferase NEP1 (Habropoda laboriosa)	302	302	89%	3.00E-99	59%
	hypothetical protein LOTGIDRAFT_208870 (Lottia gigantea)	301	301	81%	5.00E-99	68%
	PREDICTED: ribosomal RNA small subunit methyltransferase NEP1 isoform X1 (Diaphorina citri)	300	300	82%	1.00E-98	67%
	PREDICTED: ribosomal RNA small subunit methyltransferase NEP1 (Pogonomyrmex barbatus)	300	300	82%	2.00E-98	65%
	PREDICTED: ribosomal RNA small subunit methyltransferase NEP1 (Apis florea)	299	299	82%	3.00E-98	66%
Ortho 73	PREDICTED: fucose mutarotase-like isoform X1 (Crassostrea gigas)	164	164	89%	4.00E-48	53%
	PREDICTED: fucose mutarotase-like (Limulus polyphemus)	161	161	90%	8.00E-47	53%
	PREDICTED: fucose mutarotase isoform X1 (Oryzias latipes)	157	157	90%	4.00E-45	53%
	PREDICTED: fucose mutarotase (Monodelphis domestica)	156	156	90%	8.00E-45	50%
	PREDICTED: fucose mutarotase (Xiphophorus maculatus)	155	155	90%	2.00E-44	52%
Ortho 74	RING finger protein 141 (Stegodyphus mimosarum)	160	160	86%	5.00E-43	31%
	AGAP000736-PA-like protein (Anopheles sinensis)	159	159	77%	4.00E-42	35%
	zinc finger protein (Oryctes borbonicus)	154	154	74%	4.00E-41	36%
	hypothetical protein Phum_PHUM055710 (Pediculus humanus corporis)	156	156	86%	5.00E-41	32%
	PREDICTED: RING finger protein 141-like isoform X2 (Musca domestica)	154	154	82%	6.00E-41	33%
Ortho 75	conserved hypothetical protein (Pediculus humanus corporis)	332	332	69%	2.00E-109	69%
	Ubiquitin domain-containing protein UBFD1 (Zootermopsis nevadensis)	331	331	74%	3.00E-109	65%
	PREDICTED: ubiquitin domain-containing protein UBFD1-like isoform X2 (Limulus polyphemus)	327	327	70%	7.00E-108	67%
	PREDICTED: ubiquitin domain-containing protein UBFD1-like isoform X1 (Limulus polyphemus)	322	322	70%	8.00E-106	67%
	PREDICTED: ubiquitin domain-containing protein UBFD1-like (Apis mellifera)	319	319	89%	3.00E-104	56%
Ortho 76	PREDICTED: potassium channel subfamily K member 1-like isoform X1 (Cimex lectularius)	383	383	84%	7.00E-128	56%
	PREDICTED: potassium channel subfamily K member 1-like isoform X2 (Cimex lectularius)	376	376	83%	3.00E-125	58%
	PREDICTED: potassium channel subfamily K member 1-like (Limulus polyphemus)	374	374	86%	2.00E-123	56%
	PREDICTED: potassium channel subfamily K member 6-like isoform X2 (Halyomorpha halys)	357	357	87%	2.00E-117	53%
	hypothetical protein DAPPUDRAFT_218276 (Daphnia pulex)	356	356	84%	9.00E-117	53%
Ortho 77	PREDICTED: 26S proteasome non-ATPase regulatory subunit 14 (Athalia rosae)	575	575	99%	0	88%
	PREDICTED: 26S proteasome non-ATPase regulatory subunit 14 (Fopius arisanus)	574	574	99%	0	88%
	PREDICTED: 26S proteasome non-ATPase regulatory subunit 14 (Trichogramma pretiosum)	571	571	99%	0	88%
	PREDICTED: 26S proteasome non-ATPase regulatory subunit 14 (Acromyrmex echinatior)	571	571	99%	0	88%
	PREDICTED: 26S proteasome non-ATPase regulatory subunit 14 (Nasonia vitripennis)	571	571	99%	0	88%
Ortho 78	ribosomal protein L5 (Riptortus pedestris)	453	453	97%	4.00E-157	73%
	PREDICTED: 60S ribosomal protein L5 (Tribolium castaneum)	451	451	96%	3.00E-156	72%
	PREDICTED: 60S ribosomal protein L5-like (Metaseiulus occidentalis)	448	448	97%	3.00E-155	71%
	PREDICTED: 60S ribosomal protein L5 (Ciona intestinalis)	447	447	97%	1.00E-154	71%
	60S ribosomal protein L5 (Lucilia cuprina)	444	444	96%	1.00E-153	71%
Ortho 79	hypothetical protein DAPPUDRAFT_92230 (Daphnia pulex)	165	165	92%	3.00E-50	84%
	PREDICTED: barrier-to-autointegration factor-like (Limulus polyphemus)	162	162	92%	2.00E-49	84%
	conserved hypothetical protein (Pediculus humanus corporis)	160	160	92%	3.00E-48	80%
	PREDICTED: barrier-to-autointegration factor-like (Limulus polyphemus)	159	159	92%	4.00E-48	81%

	PREDICTED: barrier-to-autointegration factor (Halyomorpha halys)	158	158	92%	2.00E-47	82%
**Ortho 80**	myosin light chain 2 (Procambarus clarkii)	215	215	77%	3.00E-67	73%
	myosin light chain (Artemia franciscana)	199	199	86%	4.00E-61	57%
	myosin light chain 2 (Antheraea pernyi)	196	196	86%	1.00E-59	61%
	Myosin regulatory light chain 2 (Papilio xuthus)	197	197	88%	1.00E-59	60%
	myosin regulatory light chain 2 (Papilio xuthus)	196	196	86%	2.00E-59	60%
**Ortho 81**	hypothetical protein DAPPUDRAFT_216285 (Daphnia pulex)	323	323	86%	7.00E-105	54%
	PREDICTED: growth hormone-inducible transmembrane protein (Tribolium castaneum)	315	315	92%	2.00E-101	51%
	hypothetical protein LOTGIDRAFT_210320 (Lottia gigantea)	312	312	77%	3.00E-100	56%
	hypothetical protein OCBIM_22018462mg (Octopus bimaculoides)	310	310	77%	2.00E-99	55%
	PREDICTED: growth hormone-inducible transmembrane protein-like (Halyomorpha halys)	306	306	95%	4.00E-98	49%
**Ortho 82**	hypothetical protein DAPPUDRAFT_189030 (Daphnia pulex)	379	379	94%	2.00E-128	66%
	PREDICTED: eukaryotic translation initiation factor 3 subunit F-like (Lingula anatina)	357	357	94%	5.00E-120	62%
	ukaryotic translation initiation factor 3 subunit, putative (Pediculus humanus corporis)	345	345	95%	3.00E-115	58%
	Eukaryotic translation initiation factor 3 subunit F-1 (Zootermopsis nevadensis)	339	339	95%	1.00E-112	57%
	PREDICTED: eukaryotic translation initiation factor 3 subunit F-like (Biomphalaria glabrata)	335	335	94%	2.00E-111	58%
**Ortho 83**	PREDICTED: 26S proteasome non-ATPase regulatory subunit 11 (Tribolium castaneum)	574	574	89%	0	71%
	hypothetical protein TcasGA2_TC012621 (Tribolium castaneum)	574	574	89%	0	71%
	uncharacterized protein Dyak_GE12293, isoform B (Drosophila yakuba)	570	570	96%	0	68%
	GE12293 (Drosophila yakuba)	570	570	97%	0	67%
	GG22404 (Drosophila erecta)	570	570	97%	0	67%
**Ortho 84**	PREDICTED: mRNA-decapping enzyme 1A (Papilio machaon)	155	155	96%	9.00E-39	30%
	hypothetical protein KGM_11333 (Danaus plexippus)	152	152	96%	8.00E-38	30%
	PREDICTED: mRNA-decapping enzyme 1B isoform X1 (Bombyx mori)	150	150	96%	5.00E-37	29%
	PREDICTED: mRNA-decapping enzyme 1B isoform X2 (Bombyx mori)	149	149	96%	1.00E-36	29%
	PREDICTED: mRNA-decapping enzyme-like protein (Cerapachys biroi)	146	146	98%	2.00E-35	29%
**Ortho 85**	PREDICTED: probable 39S ribosomal protein L45, mitochondrial (Halyomorpha halys)	318	318	86%	7.00E-103	51%
	putative 39S ribosomal protein L45, mitochondrial (Zootermopsis nevadensis)	315	315	81%	2.00E-101	53%
	PREDICTED: probable 39S ribosomal protein L45, mitochondrial (Limulus polyphemus)	313	313	82%	2.00E-100	52%
	conserved hypothetical protein (Ixodes scapularis)	310	310	73%	1.00E-99	53%
	AAEL012025-PA (Aedes aegypti)	303	303	93%	9.00E-97	46%
**Ortho 86**	RNA polymerase II subunit A C-terminal domain phosphatase SSU72 (Zootermopsis nevadensis)	327	327	96%	7.00E-111	78%
	PREDICTED: RNA polymerase II subunit A C-terminal domain phosphatase SSU72 isoform X1 (Vollenhovia emeryi)	315	315	95%	6.00E-106	76%
	PREDICTED: RNA polymerase II subunit A C-terminal domain phosphatase SSU72 isoform X2 (Orussus abietinus)	315	315	95%	8.00E-106	75%
	PREDICTED: RNA polymerase II subunit A C-terminal domain phosphatase SSU72 isoform X3 (Orussus abietinus)	314	314	95%	1.00E-105	75%
	PREDICTED: RNA polymerase II subunit A C-terminal domain phosphatase SSU72 (Megachile rotundata)	314	314	95%	1.00E-105	75%
**Ortho 87**	hypothetical protein DAPPUDRAFT_306247 (Daphnia pulex)	124	124	97%	1.00E-26	30%
	PREDICTED: mediator of RNA polymerase II transcription subunit 26 (Athalia rosae)	120	120	97%	2.00E-25	26%
	PREDICTED: mediator of RNA polymerase II transcription subunit 26-like (Ceratosolen solmsi marchali)	112	112	97%	9.00E-23	25%
	PREDICTED: mediator of RNA polymerase II transcription subunit 26-like (Lingula anatina)	111	111	97%	4.00E-22	26%
	PREDICTED: uncharacterized protein LOC106473716 (Limulus polyphemus)	107	107	97%	5.00E-21	26%
**Ortho 88**	PREDICTED: PCTP-like protein (Cimex lectularius)	282	282	84%	4.00E-91	59%
	PREDICTED: PCTP-like protein (Halyomorpha halys)	281	281	98%	1.00E-90	52%

	PCTP-like protein (Zootermopsis nevadensis)	280	280	87%	4.00E-90	55%
	PREDICTED: PCTP-like protein (Crassostrea gigas)	269	269	87%	4.00E-86	52%
	PREDICTED: PCTP-like protein (Solenopsis invicta)	269	269	82%	6.00E-86	58%
**Ortho 89**	conserved hypothetical protein (Ixodes scapularis)	315	315	97%	1.00E-106	82%
	hypothetical protein DAPPUDRAFT_303043 (Daphnia pulex)	309	309	97%	4.00E-104	80%
	n-terminal acetyltransferase complex ard1 subunit (Loa loa)	297	297	97%	2.00E-99	80%
	N-terminal acetyltransferase complex ARD1 subunit homolog, putative (Brugia malayi)	296	296	97%	3.00E-99	80%
	N-terminal acetyltransferase complex ARD1 subunit homolog, putative (Brugia malayi)	296	296	97%	3.00E-99	80%
**Ortho 90**	Fkbp13, isoform B (Drosophila yakuba)	272	272	96%	1.00E-88	62%
	uncharacterized protein Dsimw501_GD25197, isoform C (Drosophila simulans)	272	272	96%	1.00E-88	62%
	GD25197 (Drosophila simulans)	273	273	96%	1.00E-88	62%
	GM15719 (Drosophila sechellia)	272	272	96%	2.00E-88	62%
	Fkbp13 (Drosophila yakuba)	272	272	96%	3.00E-88	62%

Supplementary Table S8. Sequences, PCR product size, and melting temperature of the primers designed to complete the phylogeny.

Locus	Primer sequences		PCR Product size (nuc)	Melting Temperature
	Forward	Reverse		
CYTOB2	AATTGGAAGTGARGAAGCAGCA	TTACRGACTGAACWGATGARGCW	437	58°C
UBI4	GAAACTTCTGGGGATTGAAGCAC	ACCGGYWGGCATTTGATTGA	595	57°C
NADH19	GAT TCCWCCTCTGCTYTGGT	GAYTCTCGCTTGACCCAATCM	248	56°C
METH24	TCTTGTTCGCAGCCAAARAYT	TGGGCATGGATAMCCRTTAAG	421	54°C
RIB46	GAACCWTATGTGCCYCCMGA	GKAYACGCCACAACCCRTA	529	56°C
INN2	GCAGCTCTTTYTGGTKGACAG	AAGCGTGAAGCCCTTCTTTG	571	57°C
XAA6	GCTGAAAAAGYRTGGGTTGGA	AAKCMTGRTCTGGGATATAAGM	556	52°C
VATP10	GATCCAGCGTCCRCTTCARG	CTCCRCAACCCACGTACACA	536	58°C
GROW42	YTTRGGKCTTGGAGCMCTGT	AGGACCTCCCCARCTCAAGA	481	58°C
PROT44	AAGCAGGRATTWTGGSTYTGG	RATGGCATTTGCAGTRGTWCG	461	56°C
